# Deciphering the quality of SARS‐CoV‐2 specific T‐cell response associated with disease severity, immune memory and heterologous response

**DOI:** 10.1002/ctm2.802

**Published:** 2022-04-12

**Authors:** Alberto Pérez‐Gómez, Carmen Gasca‐Capote, Joana Vitallé, Francisco J. Ostos, Ana Serna‐Gallego, María Trujillo‐Rodríguez, Esperanza Muñoz‐Muela, Teresa Giráldez‐Pérez, Julia Praena‐Segovia, María D. Navarro‐Amuedo, María Paniagua‐García, Manuel García‐Gutiérrez, Manuela Aguilar‐Guisado, Inmaculada Rivas‐Jeremías, María Reyes Jiménez‐León, Sara Bachiller, Alberto Fernández‐Villar, Alexandre Pérez‐González, Alicia Gutiérrez‐Valencia, Mohammed Rafii‐El‐Idrissi Benhnia, Daniela Weiskopf, Alessandro Sette, Luis F. López‐Cortés, Eva Poveda, Ezequiel Ruiz‐Mateos, Virgen del Rocío Hospital COVID‐19 and COHVID‐GS Working Teams

**Affiliations:** ^1^ Clinical Unit of Infectious Diseases Microbiology and Preventive Medicine Institute of Biomedicine of Seville (IBiS) Virgen del Rocío University Hospital CSIC University of Seville Seville Spain; ^2^ Department of Medical Biochemistry Molecular Biology and Immunology School of Medicine University of Seville Seville Spain; ^3^ Pneumology Service Galicia Sur Health Research Instituto (IIS Galicia Sur) Complexo Hospitalario Universitario de Vigo SERGAS‐UVigo Vigo Spain; ^4^ Group of Virology and Pathogenesis Galicia Sur Health Research Institute (IIS Galicia Sur) Complexo Hospitalario Universitario de Vigo SERGAS‐UVigo Vigo Spain; ^5^ Infectious Diseases Unit Department of Internal Medicine Complexo Hospitalario Universitario de Vigo SERGAS‐UVigo Vigo Spain; ^6^ Center for Infectious Disease and Vaccine Research La Jolla Institute for Immunology (LJI) La Jolla California USA; ^7^ Department of Medicine Division of Infectious Diseases and Global Public Health University of California San Diego (UCSD) La Jolla California USA

**Keywords:** COVID‐19, endemic coronaviruses, IL‐2, nucleocapsid, polyfunctionality, SARS‐CoV‐2, Spike, T‐cell response

## Abstract

SARS‐CoV‐2 specific T‐cell response has been associated with disease severity, immune memory and heterologous response to endemic coronaviruses. However, an integrative approach combining a comprehensive analysis of the quality of SARS‐CoV‐2 specific T‐cell response with antibody levels in these three scenarios is needed. In the present study, we found that, in acute infection, while mild disease was associated with high T‐cell polyfunctionality biased to IL‐2 production and inversely correlated with anti‐S IgG levels, combinations only including IFN‐γ with the absence of perforin production predominated in severe disease. Seven months after infection, both non‐hospitalised and previously hospitalised patients presented robust anti‐S IgG levels and SARS‐CoV‐2 specific T‐cell response. In addition, only previously hospitalised patients showed a T‐cell exhaustion profile. Finally, combinations including IL‐2 in response to S protein of endemic coronaviruses were the ones associated with SARS‐CoV‐2 S‐specific T‐cell response in pre‐COVID‐19 healthy donors’ samples. These results could have implications for protective immunity against SARS‐CoV‐2 and recurrent COVID‐19 and may help for the design of new prototypes and boosting vaccine strategies.

## Graphical abstract

“In acute infection, combinations with only IFN‐γ were deleterious and those with IL‐2 were associated with a better course of acute infection. Additionally, mild patients had a more polyfunctional T response. In severe patients, there was increased antibody production inversely associated with the expression of combinations including IL‐2. Seven months after infection, cellular and humoral responses were present, with T‐cell response quality similar to acute infection. However, previously hospitalised subjects had higher T‐cell exhaustion. Finally, an association was found between the response to HCoV and SARS‐CoV‐2 mainly mediated by IL‐2 expression in pre‐COVID‐19 participants.”

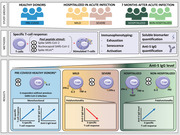

## 1 INTRODUCTION

Host immune response against Severe Acute Respiratory Syndrome Coronavirus 2 (SARS‐CoV‐2) infection is a key factor in the progression of Coronavirus Disease 2019 (COVID‐19)^1^ and its deregulation results in fatal disease in hospitalised COVID‐19 patients.^2^, ^3^ The coordination of different branches of adaptive immunity, such as CD4+, CD8+ T‐cell and antibody responses, is essential for the resolution of COVID‐19.^4^ Despite the already known role of T‐cell response against SARS‐CoV‐2 infection, there are still gaps that need to be clarified in relation to the quality of this response and its association with: (i) disease severity in acute infection, (ii) long‐lasting immune memory and (iii) the heterologous response found in healthy donors (HDs).^5^

Seminal studies in SARS‐CoV‐1 infection models showed that both CD4+^6^ and CD8+^7^ T‐cell response were involved in protection and virus clearance in acute infection. In SARS‐CoV‐2 infection, high CD4+ T‐cell response levels have been associated with mild disease and enhanced early virus clearance in acute infection, while the absence of this response was associated with fatal COVID‐19 outcome.^4^, ^8^, ^9^, ^10^ Although at a lower level of magnitude, SARS‐CoV‐2 specific CD8+ in coordination with CD4+ T‐cell response in acute infection seems to be essential for a good prognosis.^4^ Opposite to these findings, a higher magnitude and broader overall T‐cell response^11^, ^12^ and higher antibody levels against SARS‐CoV‐2^13^ have been associated with poor disease outcome. Despite all these findings, the information about the quality and polyfunctionality of T‐cell response in acute infection is scarce. The detailed and comprehensive analysis by intracellular staining (ICS) may clarify existing paradoxes about the role of T‐cell response in acute infection and may provide additional immune correlates of protection.

Equally important for immune protection and recurrent COVID‐19 is to analyse the immune memory after SARS‐CoV‐2 infection. The longevity of CD4+ T‐cell and memory B cell response against the spike protein (S) seems to be stable, while CD8+ T‐cell response lowered by half at 6–8 months after infection^14^. Moreover, it is very important to know whether the disease severity during acute infection may dictate the quality and the magnitude of long‐term immune memory. Patients with post‐acute symptoms showed a trend to decline IFN‐γ production in *N*‐specific CD8+ T‐cells 4 months after infection.^12^ However, a detailed analysis of the quality of T‐cell response at the longer term after infection is lacking. These analyses may have important implications on current vaccination strategies.

The immune memory response is not always triggered by a previous contact with SARS‐CoV‐2. Heterologous response in unexposed HDs has been found due to the sequence homology between common cold coronaviruses (HCoV) and SARS‐CoV‐2.^8^, ^15^, ^16^, ^17^ A detailed analysis of the correlation and the qualities of these responses is needed in order to know potential correlates of protection and vaccine responses.

In the present study, using an integrative approach combining antibody levels, SARS‐CoV‐2 specific CD4+ and CD8+ T‐cell response, we found specific magnitude and polyfunctionality features of this response associated with disease severity in acute infection, with long‐term immune memory in previously hospitalised and non‐hospitalised patients and also associated with heterologous response to endemic coronaviruses.

## 2 MATERIAL AND METHODS

### 2.1 Study participants

Seventy participants with confirmed detection of SARS‐CoV‐2 by reverse‐transcription polymerase chain reaction (RT‐PCR) as previously described^18^ were included. Out of these 70, 37 were hospitalised in acute phase of COVID‐19 from March 25 to May 8, 2020, while 33 participants were recruited 7 months after being diagnosed with COVID‐19, from September 9 to November 26, 2020. These participants came from the COVID‐19 patients’ Cohort Virgen del Rocio University Hospital, Seville (Spain) and the COVID‐19 Cohort IIS Galicia Sur (CohVID GS), Vigo (Spain).^19^ Thirty‐three HDs, pre‐COVID‐19 cryopreserved samples (May 12 to July 18, 2014) were included in the HD cohort, collected at Laboratory of HIV infection, Andalusian Health Public System Biobank, Seville (Spain) (C330024).^19^ Written or oral informed consent was obtained from all participants. The study was approved by the Ethics Committee of the Virgen Macarena and Virgen del Rocio University Hospital (protocol code “pDCOVID”; internal code 0896‐N‐20). Hospitalised participants during the acute phase of infection were divided in mild (*n* = 18) or severe (*n* = 19), based on the highest level of disease severity during course of hospitalization. Severe participants were those who required Intensive Care Unit admission, or having ≥6 points in the score on ordinal scale^20^ or death. The remaining acutely infected individuals by SARS‐CoV‐2 were considered mild. Blood samples were collected at a median of 3 days [interquartile range (IQR) 2.0 – 21.5] after hospitalisation and 17 days [7.0–31.5] after symptoms onset (Table S1). The group of participants discharged after infection, included previously hospitalised (*n* = 19) and previously non‐hospitalised subjects (*n* = 14). The samples from these participants were collected after a median of 201 days [180.5–221] after hospitalisation and 208 days [190–232] after symptoms onset (Table S1). Clinical and demographic data from both HD and infected subjects are described in Table S1. Acutely SARS‐CoV‐2 infected patients and COVID‐19 convalescent (previously hospitalised and not) participants were age and sex matched with HDs’ group (Table S1).

### 2.2 Cell and plasma isolation

Peripheral blood mononuclear cells (PBMCs) from HDs and participants were isolated from peripheral blood samples using BD Vacutainer® CPT™ Mononuclear Cell Preparation Tubes (with Sodium Heparin) by density gradient centrifugation at the same day of blood collection. Afterwards, PBMCs were cryopreserved in freezing medium (90% of fetal bovine serum (FBS) + 10% dimethyl sulphoxide (DMSO)) in liquid nitrogen until further use. Plasma samples were obtained using BD Vacutainer™ PET ethylenediamine tetraacetic acid (EDTA) centrifugation tubes and were cryopreserved at −80°C until further use.

### 2.3 Cell stimulation

PBMCs were thawed, washed and rested for 1 h in 0.25 μl/ml DNase I (Roche Diagnostics, Indianapolis, IN)‐containing R‐10 complete medium (RPMI 1640 supplemented with 10% FBS, 100 U/ml penicillin G, 100 l/ml streptomycin sulphate, and 1.7 mM sodium l‐glutamine). 1.5 × 10^6^ PBMCs were stimulated in vitro for 6 h with overlapping peptides of protein S (PepMix™ SARS‐CoV‐2; Spike Glycoprotein, from JPT, Berlin, Germany), 1.5 × 10^6^ with N (PepMix™ SARS‐CoV‐2; Nucleocapsid Protein, from JPT, Berlin, Germany) and 1.5 × 10^6^ with protein S of an optimised peptide pool of endemic coronavirus (SE).^21^ 1.5 × 10^6^ PBMCs incubated with the proportional amount of DMSO were included as negative control for all the samples and 1.5 × 10^6^ PBMCs stimulated with staphylococcal enterotoxin B (SEB) for each batch of experiments as a positive control. The stimulation was performed in the presence of 10 μg/ml of brefeldin A (Sigma Chemical Co, St. Louis, MO) and 0.7 μg/ml of monensin (BD Biosciences) protein transport inhibitors, anti‐CD107a‐BV650 (clone H4A3; BD Biosciences, USA) monoclonal antibody and purified CD28 and CD49d as previously described.^22^ T‐cell specific response was defined as the frequency of cells expressing intracellular cytokines and/or degranulation markers after stimulation with S, N and SE peptides, normalised with the unstimulated condition (background subtraction). The study of the specific T‐cell response to the Spike peptide pool was prioritised over that of the nucleocapsid peptide pool according to the available number of cells.

### 2.4 Immunophenotyping and intracellular cytokine staining

Both cultured PBMCs and cells for phenotypical analysis were washed (1800 rpm, 5 min, room temperature) with phosphate‐buffered saline (PBS) and incubated 35 min at room temperature (RT) with LIVE/DEAD Fixable Aqua Dead Cell Stain (Life Technologies), anti‐CD14‐BV510 (clone MφP9), anti‐CD19‐BV510 (clone SJ25C1), anti‐CD56‐BV510 (clone NMCAM16.2), anti‐CD3‐BV711 (clone SP34‐2), anti‐CD45RA‐FITC (clone L48), anti‐CD8‐APC (clone SK‐1), anti‐CD27‐APCH7 (clone M‐T271), anti‐PD‐1‐BV786 (CD279, clone EH12‐1), anti‐CD38 (clone HIT2), anti‐CD28 (clone CD28.2) (all of them from BD Bioscience); anti‐(T cell immunoreceptor with Ig and ITIM domains, TIGIT)‐PerCPCy5.5 (clone A15153G) and anti‐HLA‐DR (clone L243) (from BioLegend). PBMCs were washed with PBS and fixed and permeabilised with BD Cytofix/CytoPerm following manufacturer's protocol (Cat. No. 554714, BD Bioscience), and intracellularly stained at 4°C for 30 min with anti‐(interleukin, IL)‐2‐BV421 (clone MQ1‐17H12), anti‐(interferon, IFN)‐γ‐PE‐Cy7 (clone B27) (BD Bioscience), anti‐(tumor necrosis factor, TNF)‐α‐AF700 (clone Mab11) (BD Pharmingen), anti‐Perforin‐PE (clone B‐D48) (BioLegend). T cells were gated based on the CD3 and CD8 expression. Each subset (total memory, MEM; central memory, CM; effector memory, EM; and terminally differentiated effector memory, TEMRA) was gated based on CD45RA and CD27 expression (for gating strategy, see Figure S12). The specific T‐cell response to each stimuli was determined by the sum of the expression of each cytokine (IFN‐γ, IL‐2 and TNF‐α) in the different T‐cell subsets. To classify an individual as a responder, this value must be higher than 0.05.^22^, ^23^ Flow cytometry analyses were performed on an BD LSR Fortessa™ Cell Analyzer flow cytometer using FACS Diva software (BD Biosciences). For this analysis, at least 1 × 10^6^ events were acquired per sample and a median of 4.72 × 10^5^ live T‐cells were gated. Data were analysed using the FlowJo 10.7.1 software (Treestar, Ashland, OR).

### 2.5 Cytokine quantification

Cytokine levels were assayed in plasma samples using three different kits. sCD25 were measured by Human CD25/IL‐2R alpha Quantikine ELISA Kit (R&D System, Cat# DR2A00), using 1:2 plasma dilution; and IP‐10 by Human IP‐10 ELISA Kit (CXCL10) (Abcam, Cat# ab173194), where plasma was diluted from 1:2 to 1:4. In order to quantify IL‐6, IL‐8, IL‐1β, TNF‐α, IFN‐γ, MIP‐1α, MIP‐1β, MILLIPLEX MAP Human High Sensitivity T Cell Panel (Merck Cat# HSTCMAG‐28SK) were used, where plasma was diluted 1:2. Samples were assayed in duplicate. All of these kits were utilised according to the manufacturer's instructions.

### 2.6 Quantification of anti‐S SARS‐CoV‐2 and endemic coronaviruses IgG antibodies

Anti‐S IgG SARS‐CoV‐2 and endemic coronaviruses (NL63, OC43, 229E and HKU1) levels were measured by ELISA as previously described.^4^, ^16^, ^24^, ^25^ Briefly, Nunc Maxisorp flat‐bottomed 96‐well plates (ThermoFisher Scientific #3690) were coated with 1μg/ml of recombinant SARS‐CoV‐2 (Sino Biological, #40589‐V08B1), NL63 (Sino Biological, #40604‐V08B), OC43 (Sino Biological, #40607‐V08B), 229E (Sino Biological, #40605‐V08B) and HKU1 (Sino Biological, #40606‐V08B) Spike protein, overnight at 4°C. The following day, plates were blocked with 3% milk in PBS containing 0.05% Tween‐20 for 120 min at RT. Plasma samples were heat inactivated at 56°C for 45 min. Plasma was diluted 1:50 for endemic coronaviruses and 1:50 or 1:100 for SARS‐CoV‐2 in 1% milk containing 0.05% Tween‐20 in PBS and incubated for 90 min at RT. Plates were washed four times with 0.05% PBS‐Tween‐20. Secondary antibodies, streptavidin‐horseradish peroxidase‐conjugated mouse anti‐human IgG (Hybridoma Reagent Laboratory, Baltimore, MD, #HP6043‐HRP) was used at 1: 2,000 dilutions in 1% milk containing 0.05% Tween‐20 in PBS. Plates were washed four times with 0.05% PBS‐Tween‐20. The plates were developed using fast o‐phenylenediamine Peroxidase Substrate (Merck, #P9187), the reaction was stopped using 1M HCl, and the optical density (OD) at 490 nm (OD490) was read on a Multiskan GO Microplate Spectrophotometer (ThermoFisher Scientific) within 2 h. Two technical replicates were performed per sample. In order to validate the assays, stringent cutoff value of the SARS CoV‐2 S specific IgG signal was determined as the average of the OD of plasma samples collected from pre‐COVID HDs plus the SD multiplied by the factor 3, based on readings obtained from 21 serum samples of HDs (negative controls).^26^ The cutoff value was found to be 0.23 (mean = 0.066, SD = 0.056). Due to detectable S‐IgG levels of plasma samples from pre‐COVID HDs using the recombinant S protein from the human endemic coronaviruses, the cut‐off values for HCoV‐NL63, ‐OC43, ‐229E and ‐HKU1 ELISAs were set at arbitrary value = blank mean + 3SD). The cutoff value was found to be 0.06 (mean = 0.06, SD = 0.001) for HCoV‐NL63; 0.07 (mean = 0.06, SD = 0.003) for HCoV‐OC43; 0.09 (mean = 0.08, SD = 0.004) for HCoV‐229E and 0.07 (mean = 0.07, SD = 0.001) for HCoV‐HKU.

### 2.7 **Statistical** a**nalysis**

Non‐parametric statistical analyses were performed using Statistical Package for the Social Sciences software (SPSS 25.0; SPSS, Inc., Chicago, IL), RStudio Version 1.3.959 and GraphPad Prism version 8.0 (GraphPad Software, Inc.). Polyfunctionality was defined as the percentage of lymphocytes producing combinations of cytokines (IL‐2, TNF‐α and IFN‐γ), the degranulation marker CD107a and perforin (PRF). The simultaneous expression of the three cytokines, were also named as three functions, plus CD107a and/or PRF, as four and five functions, respectively. Polyfunctionality pie charts were constructed using Pestle version 1.6.2 and Spice version 6.0 (provided by M. Roederer, NIH, Bethesda, MD) and was quantified with the polyfunctionality index algorithm^27^ employing the 0.1.2 beta version of the FunkyCells Boolean Dataminer software provided by Martin Larson (INSERM U1135, Paris, France). Median and interquartile ranges were used to describe continuous variables and percentages to describe categorical variables. The ROUT method was utilised to identify and discard outliers. Differences between different groups were analysed by two‐tailed Mann–Whitney *U*‐test. The Wilcoxon signed‐rank test was used to analyse paired samples. Categorical variables were compared using the χ^2^ test or the Fisher's exact test. The Spearman test was used to analyse correlations between variables. All differences with a *P*‐value of < 0.05 were considered statistically significant.

## 3 RESULTS

### 3.1 **Hospitali**s**ed patients with acute SARS‐CoV‐2 infection showed an altered T‐cell phenotypic profile**

**FIGURE 1:**
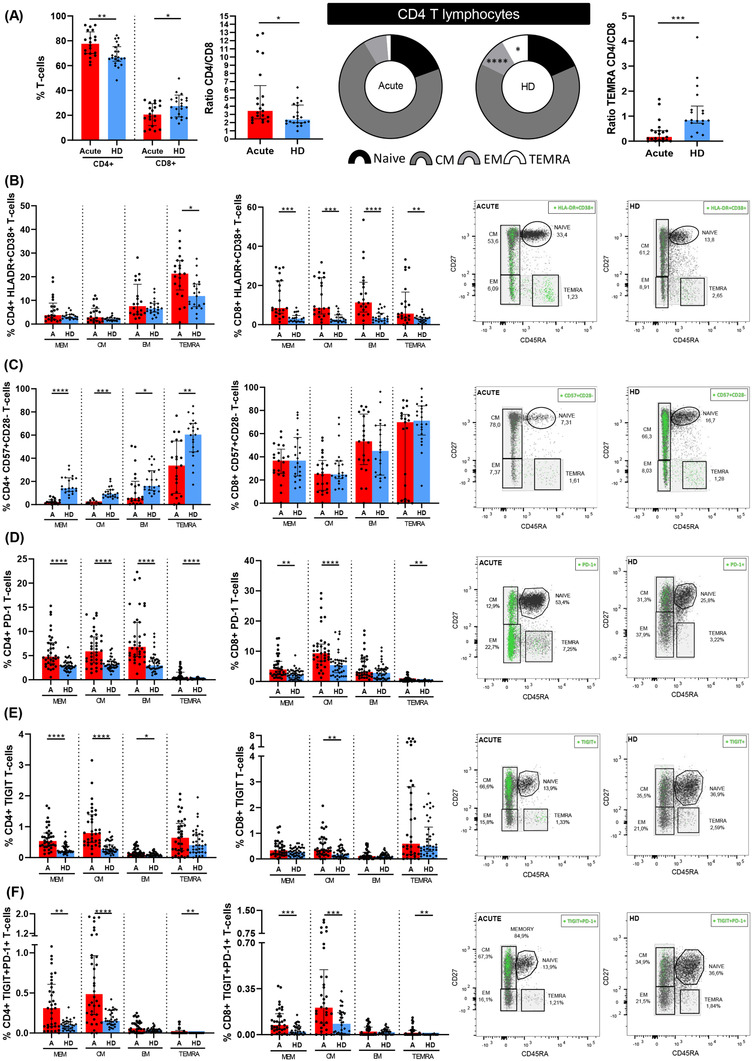
Altered CD4+ T‐lymphocyte maturation phenotype and markers of T‐cell activation, senescence and exhaustion in patients with acute SARS‐CoV‐2 infection. (A) Bar graphs representing the percentage of total CD4+ and CD8+ T cells (left panel); ratio between CD4+ and CD8+ (middle panel); and, CD4:CD8 ratio in TEMRA T‐cell subset (right panel). Pie graphs show medians of each CD4+ T‐cell subset in acute SARS‐CoV‐2 infected individual (acute) and healthy donor (HD) groups. Each subset from both groups were compared. Next bar graphs together with the representative dot‐plots of each group mentioned above show the expression of each biomarker: (B) HLA‐DR+CD38+ (activation marker); (C) CD57+CD28‐ (immune senescence marker); (D) PD‐1+; (E) TIGIT+ and (F) PD‐1+TIGIT+ T‐cells (exhaustion markers). Total memory T‐cell subset includes central memory T‐cells (CM), effector memory T‐cells (EM) and terminally differentiated effector memory T cells (TEMRA) subsets. The medians with the interquartile ranges are shown. For dot‐plots, green points are positive events of each biomarker. ROUT method was utilised to identify and discard outliers. *p < 0.05, **p < 0.01, ***p < 0.001, ****p < 0.0001. Mann–Whitney *U*‐test was used for groups’ comparisons and Spearman test for non‐parametric correlations. Categorical variables were compared using the χ2 test or the Fisher's exact test. (Acute, *n* = 37; HD, *n* = 33)

Patients hospitalised with acute SARS‐CoV‐2 infection showed higher CD4+ and lower CD8+ T‐cells levels compared with sex‐ and age‐matched pre‐COVID‐19 HDs, which resulted in higher CD4:CD8 T‐cell ratio (Figure 1A, left panel). SARS‐CoV‐2 infection was also associated with lower EM and TEMRA CD4+ T‐cell levels (Figure 1A, middle panel), while no differences were observed in CD8+ T‐cell subset levels (Figure S1A). CD4:CD8 TEMRA ratio was lower in acute COVID‐19 patients compared with HD (Figure 1A, right panel). Analyses of T‐cell activation by HLA‐DR and CD38 co‐expression revealed higher levels in all of CD8+ T‐cell subsets and TEMRA CD4+ T‐cells in SARS‐CoV‐2 infected patients (Figure 1B). This was also observed for CD38 single expression in all CD8+ T‐cell subsets (Figure S1B) but not for HLA‐DR single expression (Figure S1C,D). The levels of senescent CD4+ (CD57+CD28−), but not CD8+ T‐cell subsets, were lower in acute infection (Figure 1C). However, T‐cell exhaustion, assayed by PD‐1 and TIGIT expression and co‐expression of both markers, was higher in acute SARS‐CoV‐2 infection in most of the T‐cell subsets (Figure 1D–F).

### 3.2 **Characteristics of SARS‐CoV‐2 specific T‐cell response in acute hospitali**s**ed patients and healthy donors**

**FIGURE 2:**
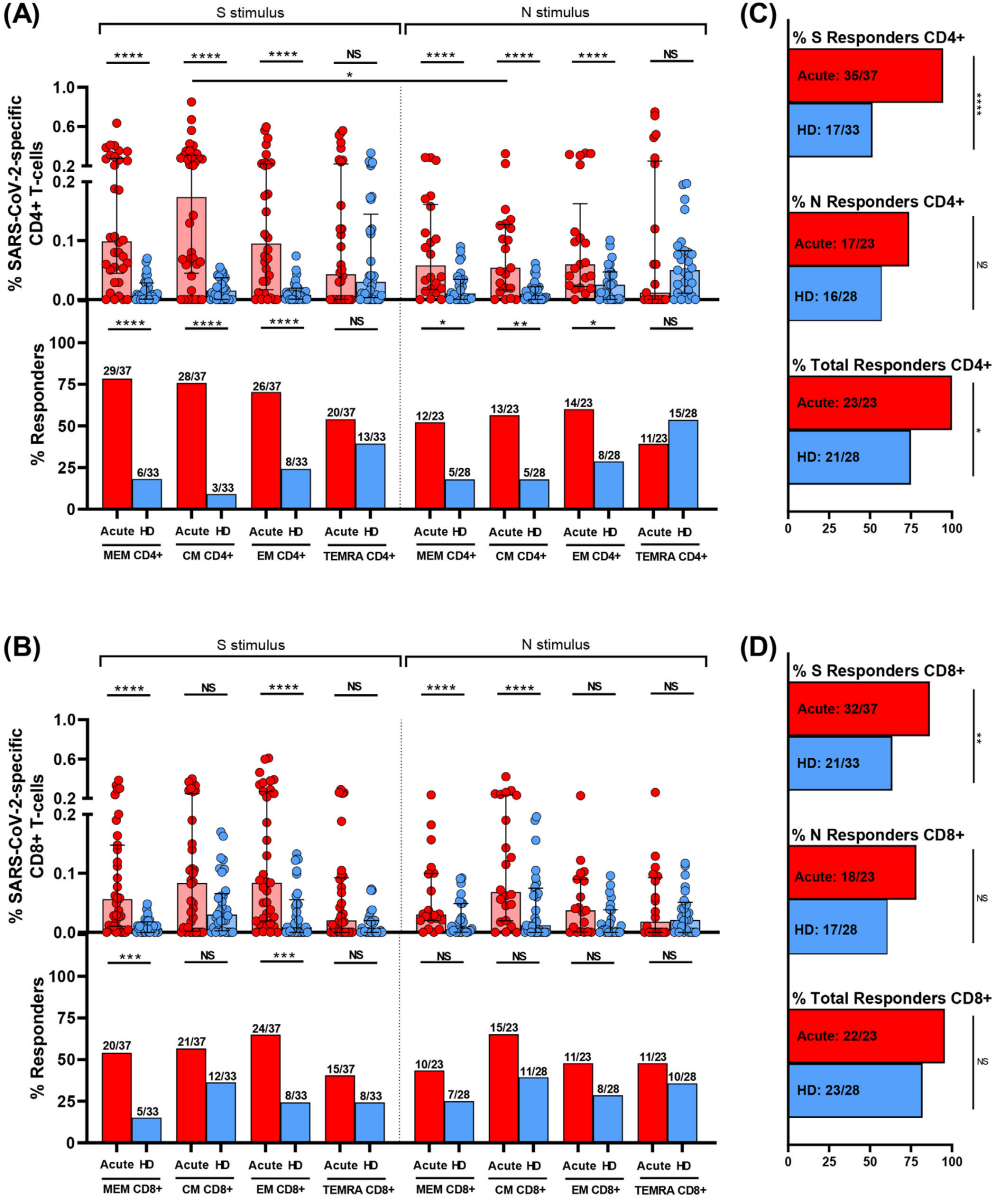
S and N specific CD4+ and CD8+ T‐cell response in acute SARS‐CoV‐2 infection individuals and healthy donors. (A,B) Bar graphs in top panels represent percentage of S and N specific CD4+ and CD8+ T‐cell response in SARS‐CoV‐2 infected patients (red) and healthy donors (blue) (upper panels). Bar graphs in low panels also show the number and percentage of responders, considering a responder subjects as those with the percentage of SARS‐CoV‐2‐specific T‐cells higher than 0.05% considering the sum of IFN‐γ, TNF‐α and IL‐2 production. (C,D) Bar graphs describe the number and percentage of responders for S peptide pool, as the sum of any CD3+CD4+ or CD3+CD8+ T‐cell subset (%S Responders); for N peptide pool, as the sum of any CD3+CD4+ or CD3+CD8+ T‐cell subset (%N responders) and the total responders as the sum of CD3+CD4+ or CD3+CD8+ S and N responses (% of total responders). The medians with the interquartile ranges are shown. Each dot represents an individual. ROUT method was utilised to identify and discard outliers. *p < 0.05, **p < 0.01, ***p < 0.001, ****p < 0.0001. Mann–Whitney *U*‐test was used for groups’ comparisons. Categorical variables were compared using the χ2 test or the Fisher's exact test. (Acute, *n* = 37; HD, *n* = 33)

We assayed SARS‐CoV‐2 specific T‐cell response by intracellular cytokine staining (ICS), this technique is a well‐established method for evaluating virus‐specific T‐cell response.^22^, ^28^ ICS, despite of using a high number of cells, allowed us to get more information about several cytokines to assay the magnitude and quality of the T‐cell response. We assessed CD4+ (CD3+CD8−) and CD8+ T‐cell response specific to spike (S) and nucleocapsid (N) peptide pools. The specific T‐cell response to each stimuli was determined by the sum of the expression of each assayed cytokine (IFN‐γ, IL‐2 and TNF‐α). To classify an individual as a responder, we consider a threshold higher than 0.05%, as previously published.^22^ First, as expected, we observed a higher magnitude of the response in most of T‐cell subsets for both peptide pools (N and S) in hospitalised acute SARS‐CoV‐2 infected patients (acute) compared to HD samples (Figure 2A,B, top panels). However, there were differences neither in the magnitude of the response nor in the proportion of responders in the TEMRA subset for both CD4+ and CD8+ T‐cells and for S and N stimuli (Figure 2A,B). In fact, there were no differences in the levels of responders for all CD8+ T‐cell subsets for N peptides (Figure 2B, bottom panels). Overall, 75% and 82% of HD had SARS‐CoV‐2 specific CD4+ and CD8+ T‐cell response, respectively, considering S+N peptides and all T‐cell subsets (Figure 2C,D).

Second, comparing the response to S and N peptide pools, there was a higher magnitude of response to S compared to N stimulus in CM CD4+ T‐cell (*p* = 0.042) and a trend in MEM CD4+ T‐cells (*p* = 0.126) (Figure 2A, top panels), however there were no differences for CD8+ T‐cell subsets (Figure 2B, top panels). Additionally, a cumulative SARS‐CoV‐2‐specific T‐cell measurement was calculated as the sum of the S and N responses (Figure S2). Our data show that all the patients had detectable SARS‐CoV‐2 specific T‐cell response considering together the response against S and N peptide pools and to all the CD4+ and CD8+ T‐cell subsets (Figures 2C,D, Figure S2).

Finally, when comparing CD4+ and CD8+ T‐cell response in hospitalised patients (acute), the magnitude of SARS‐CoV‐2 specific MEM CD4+ T‐cell response (Figure 2A, top panel) was higher compared MEM CD8+ T‐cell response (Figure 2B, top panel) for protein S (*p* = 0.048), but not different for the rest of subsets and for protein N (Figure 2A,B, top panels). In the same way, there was a higher percentage of responders for MEM CD4+ T‐cells compared to MEM CD8+ T‐cells in S protein (*p* = 0.025), there were no differences in the proportion of responders for the rest of subsets for S and N proteins (Figure 2A,B, bottom panels).

### 3.3 IFN‐γ and IL‐2 polyfunctional response in S‐specific CD4+ T‐cells was differentially associated with disease severity while IL‐2 production in S‐specific CD8+ T‐cells was associated with mild disease

**FIGURE 3:**
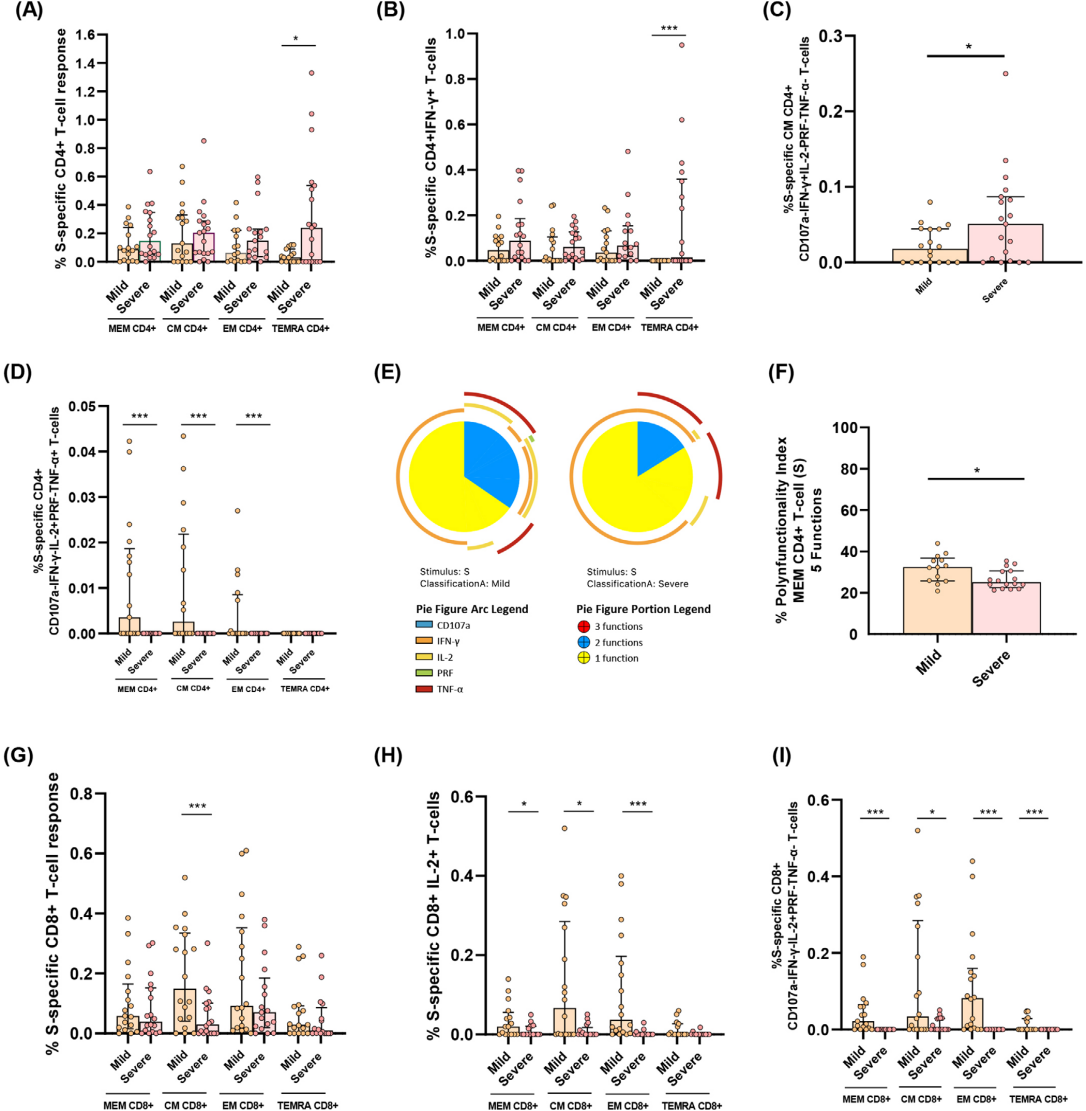
S‐specific CD4+ and CD8+ T‐cell response is associated with disease severity in acute SARS‐CoV‐2 infection. (A) Bar graphs show S‐specific CD4+ T‐cell response, considering the sum of IFN‐γ, TNF‐α and IL‐2 production, in the different CD4+ T‐cell subsets, in mild and severe acute patients’ groups. (B) S‐specific CD4+ T‐cell response considering the levels of cells producing IFN‐γ. (C) S‐specific CM CD4+ T‐cell levels of combinations only including IFN‐γ+ cells for five (IFN‐γ, TNF‐α, IL‐2, CD107a and PRF) functions. (D) S‐specific CD4+ T‐cell levels in the different T‐cell subsets of combinations including IL‐2+ and TNF‐α+ cells for five (IFN‐γ, TNF‐α, IL‐2, CD107a and PRF) functions. (E) S‐specific MEM CD4 T‐cell polyfunctionality pie charts. Each sector represents the proportion of S‐specific CD4 T‐cells producing two (blue) and one (yellow) function. Arc represents the type of function (IFN‐γ, TNF‐α, IL‐2, CD107a and PRF) expressed in each sector. Permutation test, following the Spice version 6.0 software was used to assess differences between pie charts. (F) Polyfunctional index bar graph of S‐specific MEM CD4+ polyfunctionality, for five functions. (G) Bar graphs show S‐specific CD8+ T‐cell response, considering the sum of IFN‐γ, TNF‐α and IL‐2 production, in the different CD8+ T‐cell subsets, in mild and severe acute patients’ groups, (H) S‐specific CD8+ T‐cell response considering the levels of cells producing IL‐2 and (I) S‐specific CD8+ T‐cell levels of combinations only including IL‐2+ cells for five (IFN‐γ, TNF‐α, IL‐2, CD107a and PRF) functions. The medians with the interquartile ranges are shown. Each dot represents a patient. ROUT method was utilised to identify and discard outliers. *p < 0.05, **p < 0.01, ***p < 0.001. Mann–Whitney *U* test was used for groups’ comparisons. (Mild, *n* = 18; severe, *n* = 19)

We next analysed in acute infection the association of S‐specific CD4+ T‐cell response with disease severity in hospitalised patients segregated as mild and severe patients. The S‐specific CD4+ T cell response was significantly higher in the TEMRA CD4+ T‐cell subset in severe compared to mild patients (Figure 3A). When individual cytokine production was analysed, these higher levels were attributed to IFN‐γ production in S‐specific TEMRA CD4+ subset (Figure 3B; Figure S3A), but not for IL‐2 or TNF‐α (Figure S3B). Multiple combination of cytokines, together with CD107a and perforin expression revealed that combinations only including IFN‐γ+ CM (Figure 3C, Figure S3C) and TEMRA cells (Figure S3D) were increased in severe compared with mild patients. The same occurred for combinations including IFN‐γ+ and TNF‐α+ CM cells (Figure S3E). However, combinations including IL‐2, such as IL‐2+TNF‐α+ MEM cells were increased in mild compared to severe patients (Figure 3D). In fact, S‐specific MEM CD4+ T‐cell polyfunctionality was higher in mild patients, mainly because of increased bi‐functional combinations including IL‐2 (IL‐2+TNF‐α+, IL‐2+IFN‐γ+ and IL‐2+perforin+) that were not present in severe patients, where IFN‐γ+TNF‐α+ combination was predominant (Figure 3E). It is also important to highlight that perforin expression was higher in MEM and EM subsets of mild patients in comparison with severe patients (Figure S3F). This was reflected in a higher S‐specific MEM polyfunctional index in mild compared to severe patients (Figure 3F). Additionally, we observed that the bulk of S‐specific CD4+ T‐cell response in the different subsets was inversely associated with different inflammatory markers (Figure S4A,B, Table S2), while specific combinations including IFN‐γ were directly associated with plasmatic IP‐10 levels (Figure S4A–C, Table S2). Overall, a high polyfunctional S‐specific CD4+ T‐cell response biased to IL‐2 production was associated with mild disease, while combinations only including IFN‐γ were associated with severe disease outcomes.

In relation to S‐specific CD8+ T‐cells, the bulk of CM CD8+ T‐cell response was higher in mild compared to severe patients (Figure 3G). We observed that the cytokine responsible of these differences was IL‐2, which presented higher levels in MEM, CM and EM S‐specific CD8+ T‐cells in mild subjects (Figure 3H; Figure S5A), while very low levels and no differences were observed in IFN‐γ+ and TNF‐α+ production (Figure S5B). These results were confirmed by combinations only including IL‐2 and with no expression of the rest of the cytokines, CD107a and perforin, in the same subsets: MEM, CM and EM (Figure 3I). Similar results were observed for three and four functions (Figure S5C). In summary, IL‐2 production in not terminally differentiated S‐specific CD8+ T‐cells was associated with mild disease progression in hospitalised acute SARS‐CoV‐2 infected patients.

### 3.4 **Polyfunctional N‐specific CD4+ T‐cell response was associated with mild disease in acute SARS‐CoV‐2 hospitali**s**ed patients**

**FIGURE 4:**
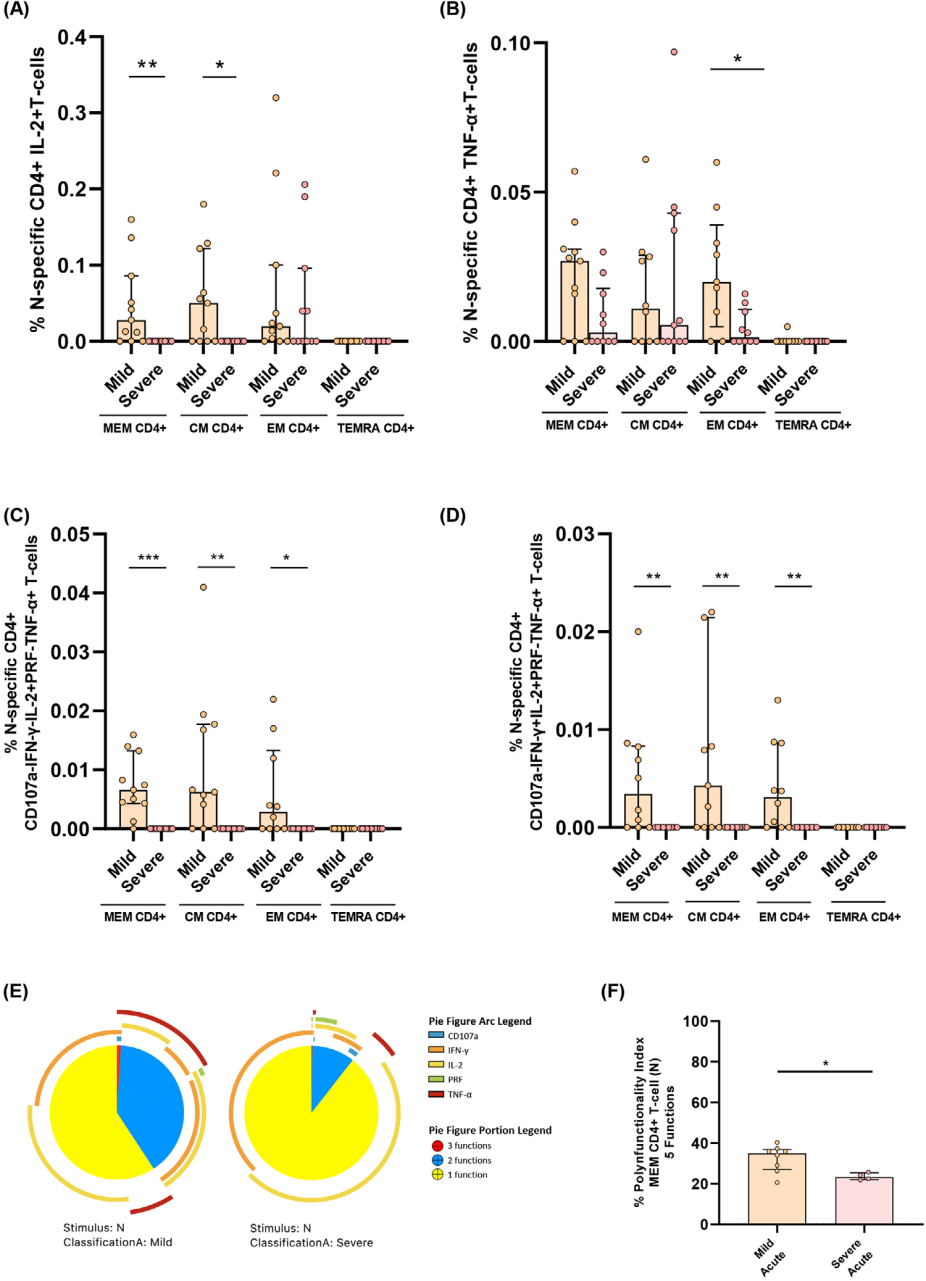
Cytokine combinations and polyfunctional N‐specific CD4+ T‐cell response are associated with COVID‐19 progression. (A) N‐specific CD4+ T‐cell response considering the levels of cells producing IL‐2 in the different CD4+ T‐cell subsets, in mild and severe acute patients’ groups. (B) N‐specific CD4+ T‐cell response considering the levels of cells producing TNF‐α in the different CD4+ T‐cell subsets, in mild and severe acute patients’ groups. (C) N‐specific CD4+ T‐cell levels in the different T‐cell subsets of combinations including IL‐2+ and TNF‐α+ cells for five (IFN‐γ, TNF‐α, IL‐2, CD107a and PRF) functions. (D) N‐specific CD4+ T‐cell levels in the different T‐cell subsets of combinations including IL‐2+, TNF‐α+ and IFN‐γ+ T‐cells for five (IFN‐γ, TNF‐α, IL‐2, CD107a and PRF) functions. (E) N‐specific MEM CD4 T‐cell polyfunctionality pie charts. Each sector represents the proportion of N‐specific CD4+ T‐cells producing three (red), two (blue) and one (yellow) function. Arc represents the type of function (IFN‐γ, TNF‐α, IL‐2, CD107a and PRF) expressed in each sector. Permutation test, following the Spice version 6.0 software was used to assess differences between pie charts. (F) Polyfunctional index bar graph of N‐specific MEM CD4+ polyfunctionality, for five functions. The medians with the interquartile ranges are shown. Each dot represents a patient. ROUT method was utilised to identify and discard outliers. *p < 0.05, **p < 0.01, ***p < 0.001. Mann–Whitney *U*‐test was used for groups’ comparisons. (Mild, *n* = 11; severe, *n* = 11)

We also analysed in detail the quality of N‐specific T‐cell response. MEM and CM IL‐2+ and EM TNF‐α+ N‐specific CD4+ T cell levels were higher in mild compared to severe patients (Figure 4A,B). We did not observe differences for the bulk of IFN‐γ+ N‐specific CD4+ T‐cell response (Figure S6A,B). Following the same profile of S‐specific CD4+ T‐cell response, combinations including only IL‐2+ and TNF‐α+ in MEM, CM and EM N‐specific CD4+ T‐cells were associated with mild disease progression (Figure 4C). Besides, we observed higher levels of combinations with triple cytokine positive MEM, CM and EM CD4+ T‐cells in mild compared to severe patients (Figure 4D). In fact, MEM N‐specific response showed a higher proportion of triple and a variety of double combinations (Figure 4E), likewise a higher MEM polyfunctional index in mild compared to severe patients (Figure 4F). These results were reproduced in polyfunctionality of CM and EM subsets with three and four functions that were also associated with mild disease progression (Figure S6C). We did not observe great differences in N‐specific CD8+ T‐cell response according with disease severity, only higher levels of combinations with only IFN‐γ+ T‐cells in severe compared to mild patients (Figure S6D).

### 3.5 **Similar magnitude of SARS‐CoV‐2 specific T‐cell response in previously hospitali**s**ed and non‐hospitali**s**ed patients seven months after infection**

**FIGURE 5:**
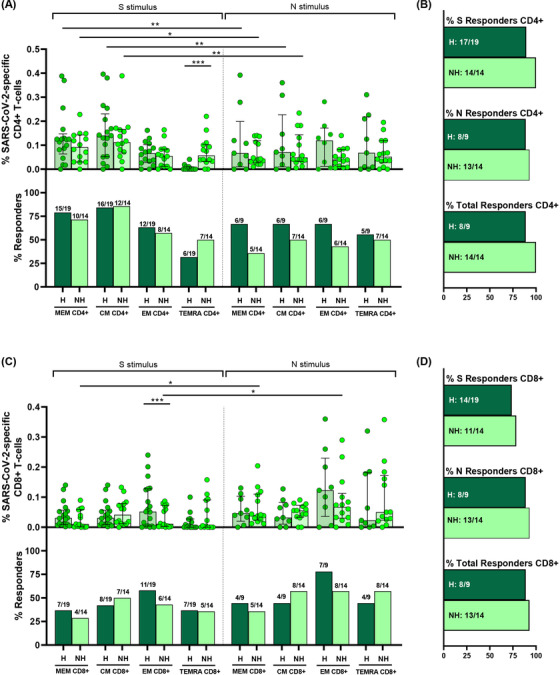
S and N specific CD4+ and CD8+ T‐cell response are present in previously hospitalised (H) and non‐hospitalised (NH) patients 7 months after SARS‐CoV‐2 infection. (A,C) Bar graphs represent percentage of S and N specific CD4+ and CD8+ T‐cell response in previously hospitalised (H) (dark green) and non‐hospitalised (NH) (light green) subjects (top panels). Bar graphs also show the number and percentage of responders, considering a responder subjects as those with the percentage of SARS‐CoV‐2‐specific T‐cells higher than 0.05% considering the sum of IFN‐γ, TNF‐α and IL‐2 production (bottom panels). (B,D) Bar graphs describe the number and percentage of responders for S peptide pool, as the sum of any CD3+CD4+ or CD3+CD8+ T‐cell subset (% S responders); for N peptide pool, as the sum of any CD3+CD4+ or CD3+CD8+ T‐cell subset (% N responders) and the total responders as the sum of CD3+CD4+ or CD3+CD8+ S and N responses (% of total responders). The medians with the interquartile ranges are shown. Each dot represents an individual. ROUT method was utilised to identify and discard outliers. *p < 0.05, **p < 0.01, ***p < 0.001, ****p < 0.0001. Mann–Whitney *U*‐test was used for groups’ comparisons. Categorical variables were compared using the χ2 test or the Fisher's exact test. (H, *n* = 19; NH, *n* = 14)

In addition to the analyses in the acute phase, we analysed the magnitude of SARS‐CoV‐2 specific T‐cell response seven months after SARS‐CoV‐2 infection in two group of individuals: (i) those previously hospitalised during acute infection and (ii) without previous hospitalisation. First, we analysed CD4+ T‐cell response. No differences were observed in the magnitude of N‐ and S‐specific T‐cell response, except for higher S‐specific TEMRA CD4+ T‐cell levels in non‐hospitalised patients compared to previously hospitalised patients (Figure 5A, top panel). Despite the 18% lower frequency of TEMRA CD4+ T‐cells in previously hospitalised responders, this difference was not statistically significant (Figure 5A, bottom panel). No differences in the proportion of responders were observed in the rest of subsets and neither for S‐ nor N‐protein (Figure 5A, bottom panel). Considering the cumulative SARS‐CoV‐2‐specific CD4+ T‐cell response (sum of the S and N response, Figure S7A) in all the T‐cell subsets, all patients, with the exception of one in the group of previously hospitalised patients, had detectable CD4+ T‐cell response (Figure 5B). We also compared the magnitude of S‐ versus N‐specific CD4+ T‐cell response in both groups. We found that the magnitude of MEM and CM response was higher in S compared to N in both, previously hospitalised (*p* = 0.008; *p* = 0.008, respectively) and non‐hospitalised patients (*p* = 0.014; *p* = 0.009, respectively) 7 months after SARS‐CoV‐2 infection (Figure 5A, top panel). Second, we analysed the magnitude of SARS‐CoV‐2 specific CD8+ T‐cell response and we found that previously hospitalised patients presented higher S‐specific EM T‐cells compared to non‐hospitalised patients (Figure 5C, top panel). There were no differences for the rest of subsets or stimuli between both groups (Figure 5C, top panel). We also did not find differences in the percentage of responders (Figure 5C, bottom panel). The analysis of the cumulative SARS‐CoV‐2‐specific CD8+ T‐cell response (Figure S7B) of previously hospitalised and non‐hospitalised, showed that 88.9% and 92.8%, respectively, had detectable SARS‐CoV‐2‐specific CD8+ T‐cell response considering all T‐cell subsets and S or N stimuli (Figure 5D). Furthermore, we found that the magnitude of MEM and EM response was higher in N compared to S peptides in non‐hospitalised patients (*p* = 0.026; *p* = 0.024, respectively), while no differences were found in previously hospitalised patients 7 months after SARS‐CoV‐2 infection (Figure 5C). Finally, we compared the magnitude of response between CD4+ and CD8+ T‐cells. We found that in both groups, MEM and CM S‐specific CD4+ T‐cell response was higher than in CD8+ T‐cells (*p* = 0.005 and *p* = 0.036 for previously hospitalised; *p* = 0.004 and *p* = 0.013 for non‐hospitalised, respectively), while no differences were found for N stimulus (Figure 5A–C).

### 3.6 **Higher CD4+TIGIT+ T‐cell and differential quality of S‐specific CD4+ CM T‐cell levels in previously hospitali**s**ed compared to non‐hospitali**s**ed patients** 7 **months after infection**

**FIGURE 6:**
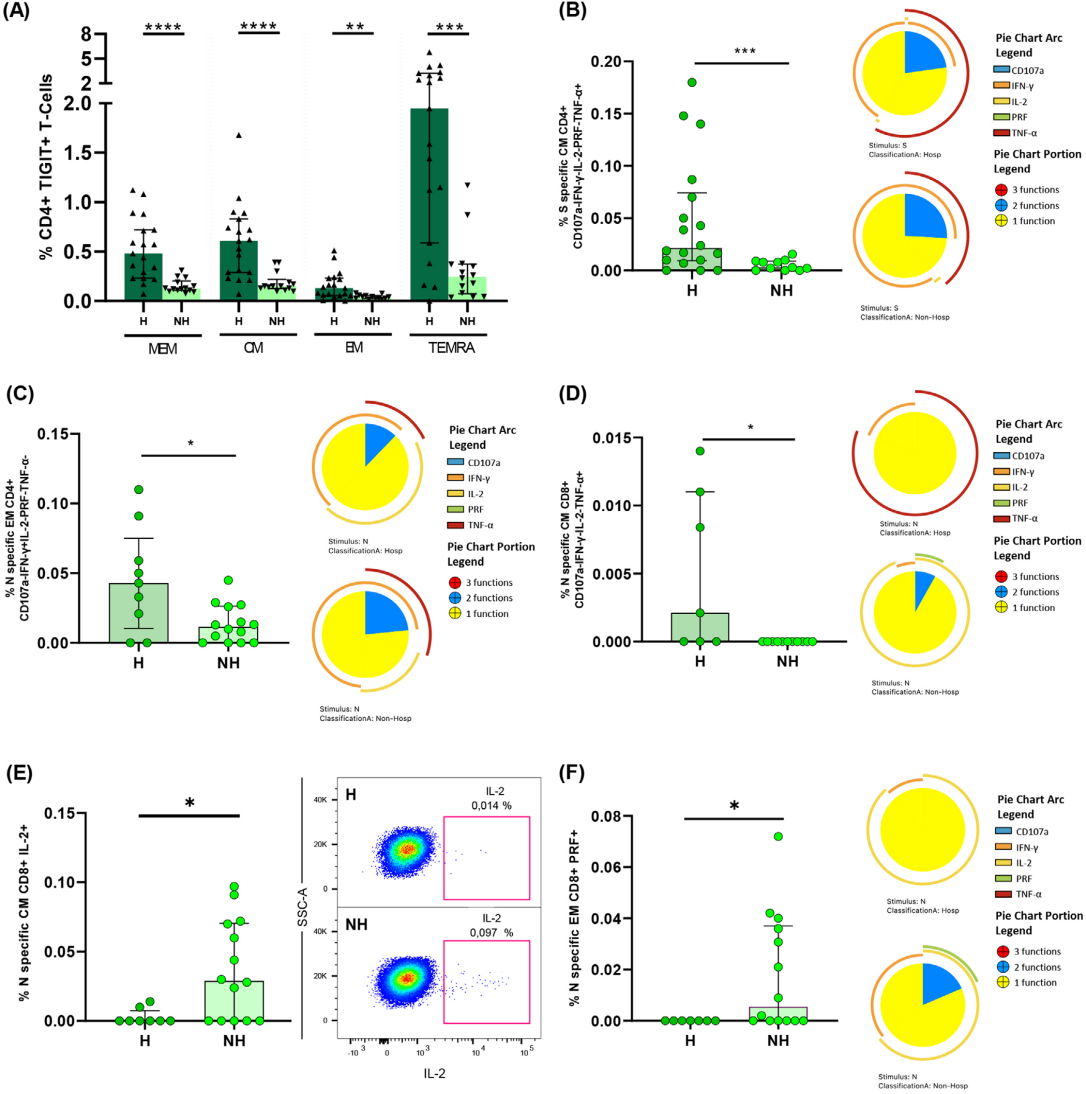
Previously hospitalised subjects during acute infection showed higher TIGIT+ CD4+ T‐cell levels and lower polyfunctional S‐ and N‐specific T‐cell response than non‐hospitalised subjects 7 months after SARS‐CoV‐2 infection. (A) TIGIT expression in each CD4+ T‐cell subset in previously hospitalised and non‐hospitalised subjects 7 months after SARS‐CoV‐2 infection. (B) S‐specific CM CD4+ T‐cell levels of combinations only including TNF‐α+ cells for five (IFN‐γ, TNF‐α, IL‐2, CD107a and PRF) functions (left panel). S‐specific CM CD4 T‐cell polyfunctionality pie charts (right panel). (C) N‐specific EM CD4+ T‐cell levels of combinations only including IFN‐γ+ cells for five (IFN‐γ, TNF‐α, IL‐2, CD107a and PRF) functions (left panel). N‐specific EM CD4 T‐cell polyfunctionality pie charts (right panel). (D) N‐specific CM CD8+ T‐cell levels of combinations only including TNF‐α+ cells for five (IFN‐γ, TNF‐α, IL‐2, CD107a and PRF) functions (left panel). N‐specific CM CD8 T‐cell polyfunctionality pie charts (right panel). (E) N‐specific CM CD8+ T‐cell levels of cells producing IL‐2 (left panel). Representative dot plot showing IL‐2 production in N‐specific CM CD8+ T‐cells (right panel). (F) N‐specific EM CD8+ T‐cell levels of cells producing PRF (left panel). N‐specific EM CD8 T‐cell polyfunctionality pie charts (right panel). For all the pie charts, each sector represents the proportion of SARS‐CoV‐2‐specific T‐cells producing two (blue) and one (yellow) function. Arc represents the type of function (IFN‐γ, TNF‐α, IL‐2, CD107a and PRF) expressed in each sector. Permutation test, following the Spice version 6.0 software was used to assess differences between pie charts. Each dot represents an individual. ROUT method was utilised to identify and discard outliers. *p < 0.05, **p < 0.01, ***p < 0.001, ****p < 0.0001. Mann–Whitney *U*‐test was used for groups’ comparisons. (H, *n* = 19; NH, *n* = 14)

After finding a similar magnitude of SARS‐CoV‐2 specific T‐cell response in both groups of individuals 7 months after infection, we assayed the quality of T‐cell response and exhaustion markers in previously hospitalised and non‐hospitalised patients. The TIGIT expression in all the CD4+ T‐cell subsets were higher in previously hospitalised than in non‐hospitalised patients (Figure 6A). We did not find differences between groups in TIGIT+ CD8+ T‐cells (Figure S8A). Likewise, PD‐1 expression was similar in all T‐cell subsets in both groups (Figure S8B,C). When we analyse multiple combination of cytokines, previously hospitalised patients showed higher levels of S‐specific CM CD4+ T‐cell with combinations only including TNF‐α (Figure 6B, left panel; Figure S8D). Furthermore, S‐specific CM CD4+ T‐cell response was more polyfunctional in non‐hospitalised patients compared with those previously hospitalised (Figure 6B, right panel). In the same line, N‐specific T‐cell response also contained higher levels of combinations only including IFN‐γ for EM CD4+ T‐cells and only including TNF‐α for CM CD8+ T‐cells in previously hospitalised than in non‐hospitalised patients (Figure 6C,D; Figure S8E,F). However, non‐previously hospitalised convalescent patients showed N‐specific CM and EM CD8+ T‐cells with higher production of IL‐2 (Figure 6D,E, right panel) and perforin (Figure 6F), respectively.

### 3.7 Anti‐S IgG levels were associated with disease severity and differentially with SARS‐CoV‐2‐specific T‐cell response in acute and convalescent subjects seven months after infection

**FIGURE 7:**
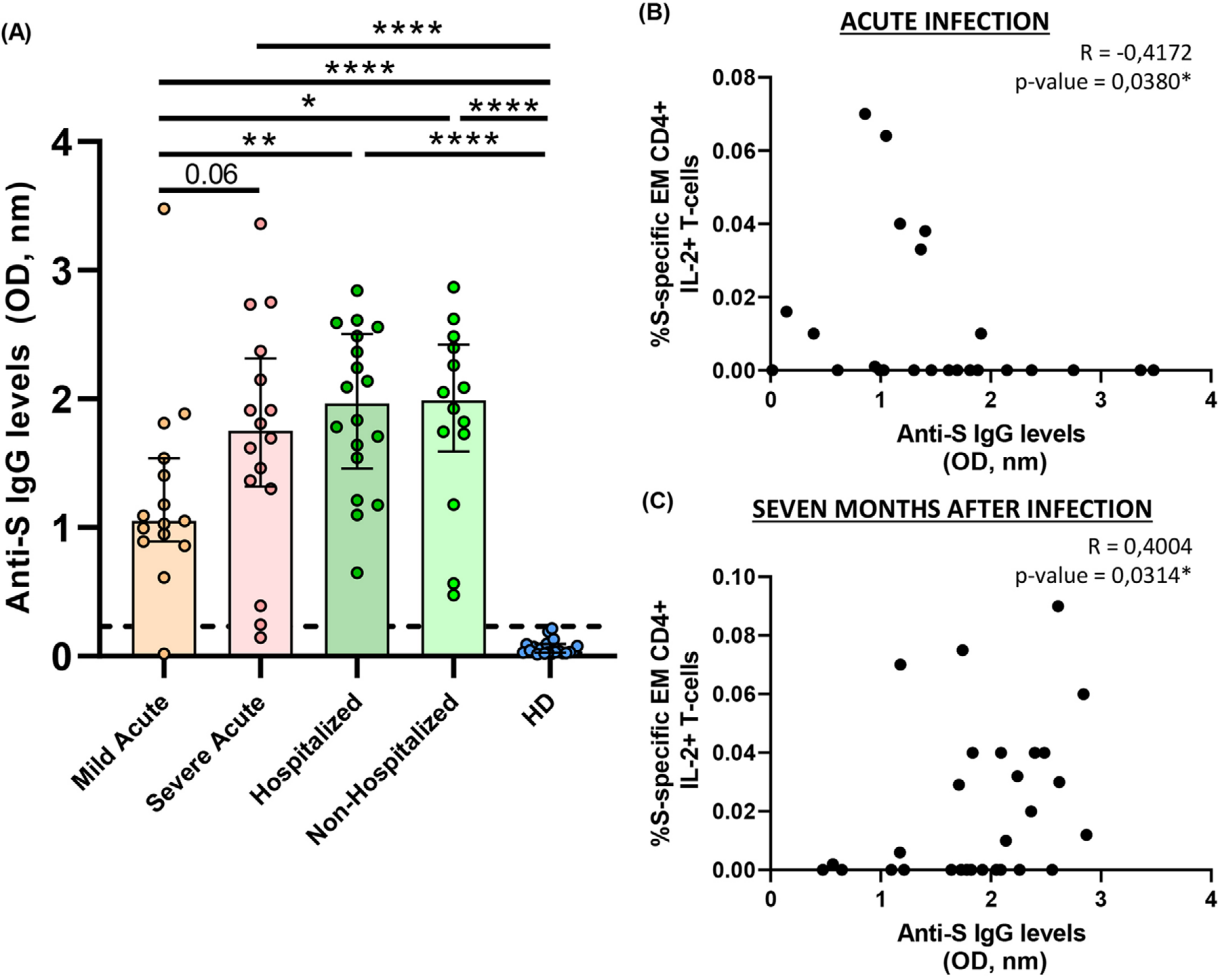
Anti‐S IgG levels are associated with COVID‐19 severity and correlate with S‐specific T‐cell response. (A) Bar graphs represent anti‐S IgG levels in each study group. The dotted line indicates the cut‐off value (0.23 OD, nm). (B) Correlation graphs between anti‐S IgG levels and the percentage of S‐specific EM CD4+ IL‐2+ T‐cell response in acute infection. (C) Correlation graphs between anti‐S IgG levels and the percentage of S‐specific EM CD4+ IL‐2+ T‐cell response previously hospitalised patients 7 months after SARS‐CoV‐2 infection. Plasma sample was used at 1:100 dilution. Each dot represents an individual. ROUT method was utilised to identify and discard outliers. *p < 0.05, **p < 0.01, ***p < 0.001, ****p < 0.0001. Mann–Whitney *U*‐test was used for groups’ comparisons and Spearman test for non‐parametric correlations. (Mild acute, *n* = 15; severe acute, *n* = 16; hospitalised, *n* = 18; non‐hospitalised, *n* = 14, HD, *n* = 21)

Next, we analysed antibody levels against S protein and the association of this humoral response with disease severity and T‐cell immunity. In acute infection, we observed a trend to increased antibody levels in severe compared to mild patients (Figure 7A). Seven months after SARS‐CoV‐2 infection anti‐S IgG levels remained high, similar to severe patients in acute infection and at higher levels compared to mild patients in both previously hospitalised and in non‐hospitalised patients (Figure 7A). As expected, all the groups had higher antibody levels compared to HD (Figure 7A). In relation to T‐cell response, in general, SARS‐CoV‐2 specific T‐cell response was inversely associated with anti‐S IgG levels in acute infection (Figure S9A), while a direct correlation was observed 7 months after infection (Figure S9B). A representative example was the moderated inverse correlation of S‐specific EM CD4+ T‐cell producing IL‐2 in acute infection (Figure 7B) compared to the direct correlation found 7 months after infection (Figure 7C).

### 3.8 Heterologous SARS‐CoV‐2 response was associated with the magnitude and the quality of endemic coronavirus response

**FIGURE 8:**
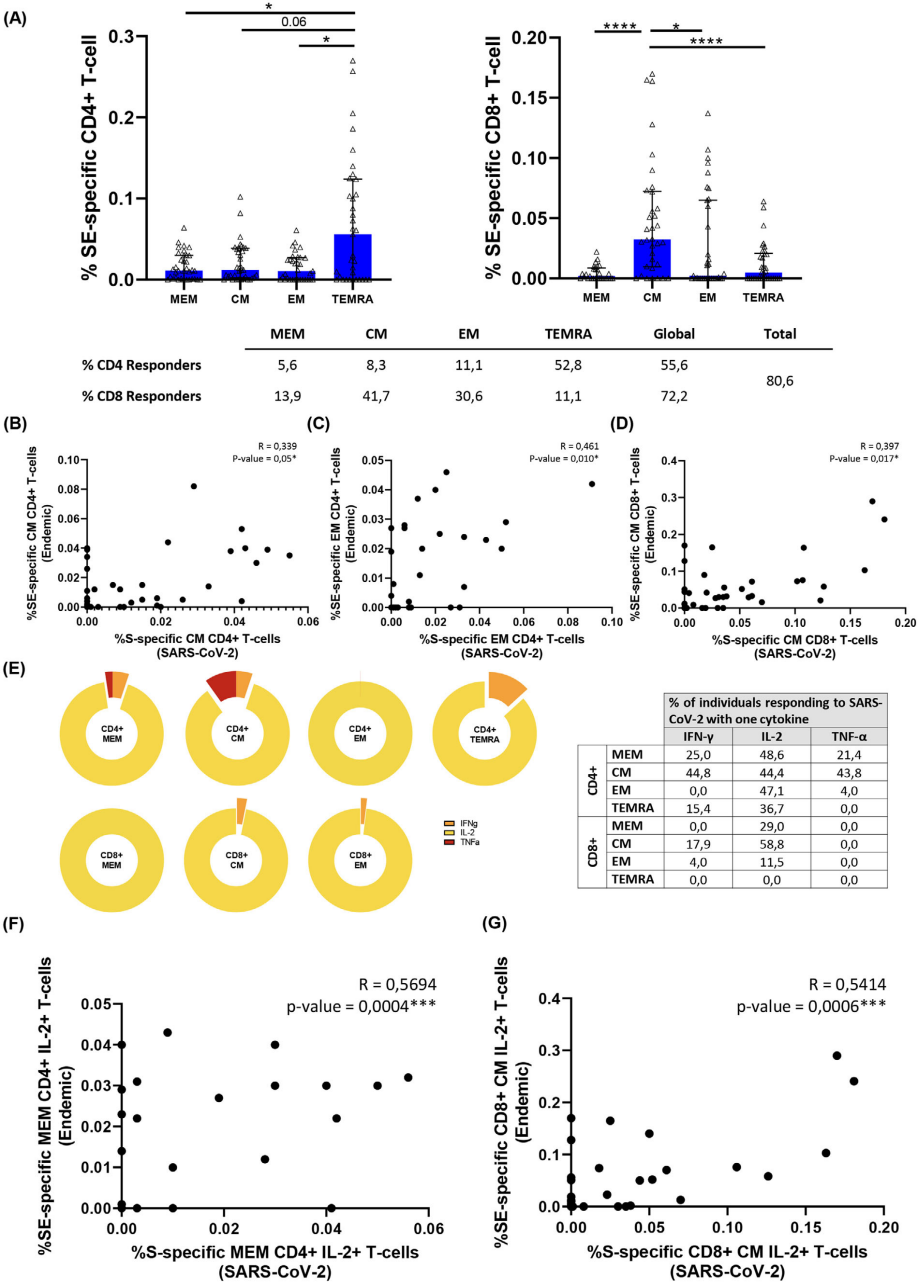
Characteristics of the cross‐reactive T‐cell response quality between SARS‐CoV‐2 and endemic coronaviruses. (A) Bar graph represents the SE‐specific T‐cell response in each CD4+ (left panel) and CD8+ (right panel) T‐cell subsets. The table shows the percentage of responders considering a responder subjects as those with the percentage of SE‐specific T‐cells higher than 0.05% considering the sum of IFN‐γ, TNF‐α and IL‐2 production. (B) Correlation between S‐Specific and SE‐specific CM CD4+ T‐cell levels. (C) Correlation between S‐Specific and SE‐specific EM CD4+ T‐cell levels and (D) correlation between S‐Specific and SE‐specific CM CD8+ T‐cell levels in healthy donors. (E) Pie graphs represent IFN‐γ, IL‐2 and TNF‐α expression in each T‐cell subset, where median percentages of this expression are shown in right table. (F) Correlation between S‐Specific and SE‐specific MEM CD4+ IL‐2+ T‐cells and (G) S‐specific and SE‐specific CM CD8+ IL‐2+ T‐cell levels. Each dot represents an individual. ROUT method was utilised to identify and discard outliers. *p < 0.05, **p < 0.01, ***p < 0.001, ****p < 0.0001. Mann–Whitney *U*‐test was used for groups’ comparisons and Spearman test for non‐parametric correlations. (HD, *n* = 33)

Similar to previously reported,^21^ we found that a high percentage of HD (pre‐COVID‐19 samples) presented detectable CD4+ and CD8+ SARS‐CoV‐2 specific T‐cell response (75% and 82%, respectively, Figure 2). In order to characterise this immune response, we performed anti‐S IgG levels and specific T‐cell response by ICS using an optimised peptide pool for the four human endemic coronaviruses^21^. In acute SARS‐CoV‐2 infected participants we observed a direct correlation of anti‐S SARS‐CoV‐2 IgG levels with those of three out of the four endemic coronaviruses (HCoV‐NL63, ‐OC43 and ‐HKU1) (Figure S10, Table S3). When we split this group, we only found a positive correlation of anti‐S SARS‐CoV‐2 IgG and anti‐S HCoV‐NL63 and ‐OC43 levels in severe (Figure S10, Table S3) but no correlation was found in mild patients (Figure S10, Table S3). In HD, we also found a positive correlation of anti‐S SARS‐CoV‐2 IgG and anti‐S HCoV‐OC43, ‐229E and HKU‐1 levels (Figure S10, Table S3). Finally, in all the groups together, anti‐S SARS‐CoV‐2 IgG levels were directly associated with anti‐S IgG levels of the beta‐coronaviruses HCoV‐OC43 and ‐HKU1 (Figure S10, Table S3). After that, we performed S‐specific T‐cell response to the optimised peptide pool of endemic coronaviruses (SE) in HD. We found detectable SE T‐cell response in all the CD4+ and CD8+ T‐cell subsets (Figure 8A). Analysing the bulk of SE T‐cells reported higher levels of response in the TEMRA and CM subsets, in CD4+ and CD8+ T‐cells, respectively. Overall, the proportion of responders was 55.6% for CD4+ and 72% for CD8+ T‐cells and 80% the global response (Figure 8A). The response to human endemic coronaviruses correlated to S‐specific for SARS‐CoV‐2 in CM and EM CD4+ subsets (Figure 8B,C) and in CM CD8+ subset (Figure 8D). Attending to the quality of this response, it was mainly monofunctional and IL‐2 production prevailed in CD4+ and CD8+ T‐cells respect to other cytokines (Figure 8E; Figure S11A). Interestingly, combinations including IL‐2, but not IFN‐γ, in response to human endemic coronaviruses correlated with S‐specific SARS‐CoV‐2 response, for CD4+ MEM (Figure 8F), CM and EM subsets (Figure S11B,C) and CD8+ CM T‐cells (Figure 8G).

## 4 DISCUSSION

In the present study, analysing 103 subjects, we describe features of SARS‐CoV‐2 specific humoral and T‐cell response differentially associated with disease severity in hospitalised patients during acute infection. This response is long‐lasting seven months after infection independently whether patients were previously hospitalised or not, although previous hospitalisation was associated with exhausting T‐cell features present in acute infection. Finally, we comprehensively analysed the features of the high levels of cross‐reactive response between SARS‐CoV‐2 and human endemic coronaviruses in HDs.

We used ICS for the systematic analysis of SARS‐CoV‐2 specific T‐cell response. ICS is a technique commonly used for analysing T‐cell response against viral infections^22^, ^28^ and can be complementary to other strategies as T‐cell receptor dependent activation induced marker (AIM).^16^, ^29^, ^30^ Although a high amount of cells is needed, a comprehensive cytokine‐dependent functional characterisation of virus specific T‐cell response can be achieved.^5^ We analysed the response against protein S and N, because these are the main targets, in terms of magnitude, of SARS‐CoV‐2 specific T‐cell response.^16^

First, we found that all patients in acute infection, independently of disease severity, had detectable SARS‐CoV‐2 specific T‐cell response, as a summation of S+N response and considering all CD4+ and CD8+ T‐cell subsets. These data were remarkable based on the high activation and inhibitory receptor T‐cell levels found in the present study in response to SARS‐CoV‐2 infection. Patients in acute infection showed low CD8+ T‐cell levels and consequently high CD4+:CD8+ T‐cell ratio compared to HD together with high T‐cell inhibitory receptor levels, such as high levels of PD‐1, as previously reported,^9^, ^31^ and TIGIT, likewise high levels of activation in all the CD8+ T‐cell subsets but only for terminally differentiated CD4+ T‐cells. This scenario was compatible with a prominent T‐cell migration to damaged tissue^32^, ^33^ which was associated with lower TEMRA CD4+:CD8+ T‐cell ratio, because of high levels of TEMRA CD4+ HLA‐DR+CD38+ T‐cells accompanied by low peripheral CD4+ T‐cell senescent levels, pointing out to preferential tissue recruitment of these cells.

According with previous studies, we found higher levels of response in CD4+ compared to CD8+ T‐cells and against S than N protein in CD4+ T‐cells.^31^ This was confirmed in the MEM subset. The higher CD4+ compared to CD8+ T‐cell response levels may be due to the use of optimised peptide pools for MHC‐II. However, higher CD4+ compared to CD8+ T cell response levels have been traditionally associated with control of SARS‐CoV‐1 infection.^6^, ^34^ In our cohort, the high number of responders could be mainly due to CD8+ T‐cells (96%), while other cohorts only found 53% of responders in this subset.^4^ In this sense, the use of ICS for assaying T‐cell response provides more information about the expression of other cytokines than IFN‐γ in comparison to other methods.

In acute infection, some studies have associated a deleterious effect of SARS‐CoV‐2 specific T‐cell response with disease progression,^11^, ^12^ while others have shown a beneficial role associated with mild disease in acute hospitalised patients.^9^ Results presented herein may clarify this paradox. Severe compared to mild patients showed higher IFN‐γ but lower IL‐2+TNF‐α+ S‐ and N‐specific CD4+ T‐cell levels. These results point out that studies using only IFN‐γ ELISPOT technology^11^, ^35^ would show that SARS‐CoV‐2‐specific T‐cell response in acute infection is deleterious. However, we found that combinations including IL‐2 were polyfunctional, including the cytotoxic marker perforin, pointing out to a higher antiviral activity with a classical signature of canonical Th1 cells^4^, ^5^, ^8^, ^11^ associated with mild disease in hospitalised patients. This fact was supported by S‐specific CD8+ T‐cell response, which mainly consisted in IL‐2 production, in this case in monofunction, at higher levels in mild patients. Besides, it is important to highlight the higher polyfunctionality found in N compared to S protein. Polyfunctional combinations of three functions (IFN‐γ+IL‐2+TNF‐α+) were at higher levels in MEM, CM and EM CD4+ T‐cells in mild compared to severe patients in response to N protein. A successful outcome of acute disease may come for the combination and coordination of CD4+, CD8+ T‐cell response and antibody production against SARS‐CoV‐2^4^. For having an integrated picture of acute anti‐SARS‐CoV‐2 response, we also assayed anti‐S IgG levels that in accordance with previous studies,^9^, ^13^ were at higher levels in severe patients. Although overall, in acute infection, as previously reported,^9^, ^11^ S‐specific T‐cell response was directly associated with anti‐S IgG levels, we found that the production of IL‐2 by EM S‐specific T‐cells was inversely associated with antibody production. In the same line, overall S‐specific CD4+ T‐cell response was inversely associated with inflammatory markers; however, combinations including IFN‐γ were directly associated with IP‐10 plasmatic levels which has previously associated with disease progression.^36^, ^37^ Altogether, these results define two different quality profiles of humoral and S/N‐specific T‐cell response associated with diseases progression in hospitalised patients: (i) a mild disease progression profile associated with IL‐2 production, inversely correlated with anti‐S IgG levels and associated with a higher T‐cell polyfunctionality, which should promote CD4+ T‐cell proliferation and CD4+ T‐cell help to CD8+ T‐cells together with antiviral potential enabling rapid virus clearance; and (ii) a severe disease progression profile consisting in high anti‐S IgG levels and combinations only including IFN‐γ, mainly in terminally differentiated T‐cells with absence of perforin production, no CD8+ T‐cell help and limited antiviral potential what may favour the failure to early control of the virus and poor disease outcome.

Next, we sought to analyse the immune memory to SARS‐CoV‐2 7 months after infection in two groups of subjects with different course of the disease: patients that overcame the disease without the need of hospitalisation and previously hospitalised patients. We observed that in both groups, all subjects displayed detectable T‐cell response, considering S+N response and all CD4+ and CD8+ T‐cell subsets. This is in accordance with immune memory found to SARS‐CoV‐1 infection, which have been shown to last for years^38^ and agreed with the magnitude of T‐cell response found 8 months after SARS‐CoV‐2 infection in previously non‐hospitalised subjects.^14^ Although in that study using AIM they found only SARS‐CoV‐2 specific CD8+ T‐cell response in 50%, while we observed 91% of responders but 75% in previously hospitalised patients.^14^ Despite the general absence of difference in the magnitude of T‐cell response between both groups, in terms of quality of this response, previously hospitalised patients showed higher T‐cell exhaustion levels (TIGIT and PD‐1 expression) and higher S and N‐specific T‐cell levels of combinations of only including IFN‐γ and TNF‐α production compared to non‐hospitalised patients. Additionally, non‐hospitalised patients presented higher IL‐2 and perforin production in N‐specific CD8+ T‐cells compatible with a preserved antiviral activity. This profile is reminiscent of the one found in severe compared to mild patients in acute infection. However, on the contrary to what happened in acute disease, 7 months after infection in previously hospitalised subjects, anti‐S IgG levels were directly, not inversely, associated with SARS‐CoV‐2 specific T‐cell response, especially that enriched in IL‐2 production which was associated with a good prognosis in acute infection. These results demonstrate that anti‐SARS‐CoV‐2 humoral and cellular response are long‐lasting and robust at least 7 months after infection in both non‐hospitalised and previously hospitalised patients. However, previously hospitalised patients showed T‐cell exhaustion and some signs of SARS‐CoV‐2 specific T‐cell response associated with disease progression in acute infection, although this response was more IL‐2 biased, which was associated with good prognosis. This defects found in previously hospitalised patients 7 months after infection may be selectively associated with long‐COVID symptoms, as has been recently reported 4 months after infection;^12^ however, we cannot confirm it because information about long‐lasting symptoms were not recorded in this cohort as this was not the aim of the present study.

Finally, we found high levels of cross‐reactive CD4+ and CD8+ T‐cell response to SARS‐CoV‐2 (75% and 82%, respectively) in pre‐COVID‐19 HD samples. These levels were even higher than those found in previous cohorts showing 20%–50% of cross‐reactivity.^8^, ^16^, ^38^, ^39^ Using an optimise peptide pool^21^ for the four endemic coronaviruses: NL63, OC43, 229E and HKU1, we found that endemic S‐specific CD4+ and CD8+ T‐cell response was directly correlated with SARS‐CoV‐2 S‐specific CD4+ and CD8+ T‐cell response in EM and CM subsets. These results confirm cross‐reactive SARS‐CoV‐2 specific T‐cell response with endemic coronavirus. Comprehensively analyses of endemic S‐specific T‐cell response was mainly induced by TEMRA CD4+ T‐cells and CM CD8+ T‐cells and we found that the response was totally biased to IL‐2 production, what may explain some previously published results using IFN‐γ ELISPOT that did not find cross‐reactive response.^11^ In fact, combinations including IL‐2, but not IFN‐γ, were the ones associated with SARS‐CoV‐2 S‐specific CD4+ and CD8+ T‐cell response. These results suggest that this pre‐existing T‐cell memory could reduce the likelihood of suffering from COVID‐19, although this cannot be confirmed based on the present results. However, whether a different quality of endemic T‐cell specific response, based mainly in IL‐2 or IFN‐γ production, may contribute to variations in COVID‐19 progression is currently unknown.

One limitation of this study is that patients included were mostly elderly subjects (71 [62–90] years old) all recruited in the first wave of COVID‐19 in Spain, in those dates, samples were difficult to obtain and experimental therapies with very limited but transitory immunosuppressive effects were administered what may have affected the levels of immune parameters in acute infection. However, in those patients who were treated with IFN‐β and corticosteroids, samples were collected time enough after these therapies to reverse the potential effects (24 days [7–28] and 9 days [1–21], respectively) what may have not affected the results presented herein. It is important to note that despite of the different time of recruitment since hospitalisation between severe and mild patients in acute infection, the same differences in SARS‐CoV‐2 specific T‐cell parameters were found between mild patients (3 [2–3] days) and a subgroup of severe patients (2.5 [1.0–3.3] days) with the same time since hospitalisation (data not shown). According to these results, the time of sample collection is independent of the clinical phenotype (mild vs. severe). Seven‐months post‐infection, one potential bias may come from the group of hospitalised subjects that were composed by patients with previous mild (42%) or severe (58%) disease, however, as no differences were found in any parameter associated to T‐cell response (data not shown) between these two subgroups, they formed part of the same group of previously hospitalised and were compared with non‐hospitalised patients. ICS needs a notable amount of cells to be assayed, this avoids us to perform endemic virus‐specific T‐cell response in COVID‐19 samples; however, it has allowed us to obtain comprehensive data about the quality of SARS‐CoV‐2 specific T‐cell response. Finally, anti‐S IgG levels were assayed against the whole S protein and not for the Receptor Binding Protein (RBD), cross‐reactive reaction cannot be excluded and results have to be interpreted taking this into account. In the same way, further research is needed to confirm and correlate our T‐cell response profile with neutralising antibody levels and B‐cell polyfunctionality.

In summary, our results gain insights in the characteristics of T‐cell response associated with disease severity in acute infection, supporting important information about correlates of immune protection, such as a broader polyfunctional CD4+ T‐cell response with predominance of IL‐2 production also present in SARS‐CoV‐2 specific CD8+ T‐cell response, distinguished mild disease progression from severe COVID‐19 characterised by an inefficient monofunctional IFN‐γ+ CD4+ T‐cell response in acute hospitalised patients. However, independently of previous hospitalization, SARS‐CoV‐2 specific T‐cell response was robust 7 months after infection, although some defects associated with T‐cell exhaustion were observed in previously hospitalised patients.

These results could have implications for protective immunity against SARS‐CoV‐2 and recurrent COVID‐19 and may help to identify populations, apart from the classical risk ones, that are in the need of new boosting of existing vaccines, by thoroughly assessing the magnitude and quality of their specific T‐cell and humoral response to SARS‐CoV‐2, as well as for improving the design of new prototypes looking for the quality profile of the specific T‐cell response to SARS‐CoV‐2 presented herein in ongoing vaccine clinical trials in order to achieve of broader long‐lasting protection against COVID‐19.

## CONFLICT OF INTERESTS

Alessandro Sette is a consultant for Gritstone, Flow Pharma, Arcturus, Immunoscape, CellCarta, OxfordImmunotech and Avalia. LJI has filed for patent protection for various aspects of T cell epitope and vaccine design work. All other authors declare that they have no competing financial interests.

## Supporting information

Supporting information
