# Supplementary material for: Deciphering the quality of SARS‐CoV‐2 specific T‐cell response associated with disease severity, immune memory and heterologous response
Source: Clin Transl Med. 2022 Apr 12;12(4):e802. doi: 10.1002/ctm2.802 (PMC9005926; doi:10.1002/ctm2.802)
Supplement: Supplementary file 1 — Supporting information [file CTM2-12-e802-s001.docx]

**SUPPLEMENTARY MATERIALS**

**Deciphering the quality of SARS-CoV-2 specific T-cell response associated with disease severity, immune memory and heterologous response**

Alberto Pérez-Gómez et al.

*Corresponding author information:

Ezequiel Ruiz-Mateos Carmona, Ph.D.

Telephone: 0034 955923109

Fax: 0034 955923101

Email address: [eruizmateos-ibis@us.es](mailto:eruizmateos-ibis@us.es)

# **SUPPLEMENTARY MATERIALS**

**Supplementary Figure 1. CD8+ T-cells maturation phenotype and activation markers. (A)** Pie graphs show medians of each CD8+ T-cell subset in acute
SARS-CoV-2 infected individuals and healthy donor groups. Bar graphs represent the percentage of **(B)** CD8+CD38+, **(C)** CD4+HLA-DR+ and **(D)** CD8+HLA-DR+ T-cells. The medians with the interquartile ranges are shown. ROUT method was utilized to identify and discard outliers. *p < 0.05, **p <0.01, ***p < 0.001, ****p < 0.0001. Mann-Whitney U test was used for groups’ comparisons and Spearman test for non-parametric correlations. (Acute, n= 37; HD, n= 33)

**Supplementary Figure 2. Combined SARS-CoV-2 specific T-cell response to N and S proteins in acute SARS-CoV-2 infected individuals and healthy donors. (A)** Bar graphs represent the percentage of S plus N-specific CD4+ T-cell response. **(B)** Bar graphs represent the percentage of S plus N-specific CD8+ T-cell response. The medians with the interquartile ranges are shown. Each dot represents an individual. ROUT method was utilized to identify and discard outliers. *p < 0.05, **p < 0.01, ***p < 0.001,
****p < 0.0001. Mann-Whitney U test was used for groups’ comparisons. (Acute, n= 37; HD, n= 33)

**Supplementary Figure 3. Additional S-specific CD4+ T-cell response features associated with disease severity in acute SARS-CoV-2 infection. (A)** Representative dot-plot of expression of IFN-γ+ in CD4+ TEMRA cells. **(B)** Bar graphs represent S-specific CD4+ T-cell response considering the levels of cells producing IL-2 (left panel) and TNF-α (right panel). **(C)** S-specific CM CD4+ T-cell levels of combinations only including IFN-γ+ cells for three (IFN-γ, TNF-α and IL-2) and four (IFN-γ, TNF-α, IL-2 and CD107a) functions. **(D)** S-specific TEMRA CD4+ T-cell levels of combinations only including IFN-γ+ cells for three (IFN-γ, TNF-α and IL-2), four (IFN-γ, TNF-α, IL-2 and CD107a) and five (IFN-γ, TNF-α, IL-2, CD107a and PRF) functions. **(E)** S-specific CM CD4+ T-cell levels of combinations including IFN-γ+ and TNF-α+ cells for three
(IFN-γ, TNF-α and IL-2), four (IFN-γ, TNF-α, IL-2 and CD107a) and five (IFN-γ,
TNF-α, IL-2, CD107a and PRF) functions. **(F)** Percentage of S-specific production of: PRF+ in CD4+ total memory (MEM) and central memory (CM) T-cell subsets. The medians with the interquartile ranges are shown. Each dot represents a patient. ROUT method was utilized to identify and discard outliers.*p < 0.05, **p < 0.01.
Mann-Whitney U test was used for groups’ comparisons. (Mild, n= 18; Severe, n= 19)

**Supplementary Figure 4. S-specific T-cell response is differentially associated to inflammatory markers in acute SARS-CoV-2 infected patients**. Correlation matrix representing associations between representative parameters of S-specific CD4+ T-cell response with inflammatory markers including TNF-α, IL-6, IL-8, IL1-β, MIP1-α,
MIP1-β, IFN-γ, CD25 and IP-10, in acute SARS-CoV-2 infected patients. Blue color represents positive correlations and red color shows negative correlations. The intensity of the color indicates the R coefficient. ROUT method was utilized to identify and discard outliers. *p < 0.05, **p < 0.01, ***p < 0.001. Spearman test was used for non-parametric correlations. (Acute, n=34)

**Supplementary Figure 5. Additional features of S-specific CD8+ T-cell response associated with disease severity. (A)** Representative dot-plot of IL-2 expression in
S-specific CM CD8+ T-cells. **(B)** Bar graphs represent S-specific CD8+ T-cell response considering the levels of cells producing IFN-γ (left panel) and TNF-α (right panel). **(C)** S-specific CD8+ T-cell levels of combinations only including IL-2+ cells for three
(IFN-γ, TNF-α and IL-2 and CD107a) and four (IFN-γ, TNF-α, IL-2 and CD107a) functions. The medians with the interquartile ranges are shown. Each dot represents a patient. ROUT method was utilized to identify and discard outliers. *p < 0.05, **p < 0.01, ***p < 0.001, ****p < 0.0001. Mann-Whitney U test was used for groups’ comparisons. (Mild, n= 18; Severe, n= 19)

**Supplementary Figure 6. Additional features of N-specific T-cell response associated with disease progression. (A)** Bar graphs show N-specific CD4+ T-cell response, considering the sum of IFN-γ, TNF-α and IL-2 production, in the different CD4+ T-cell subsets, in mild and severe acute patients’ groups, **(B)** N-specific CD4+ T-cell response considering the levels of cells producing IFN-γ. **(C)** N-specific CM and EM CD4 T-cell polyfunctionality pie charts for three and four functions. Each sector represents the proportion of N-specific CD4 T-cells producing three (red), two (blue) and one (yellow) function. Arcs represents the type of function (IFN-γ, TNF-α, IL-2, CD107a and PRF) expressed in each sector. Permutation test, following the Spice version 6.0 software was used to assess differences between pie charts. **(D)** N-specific EM CD8+ T-cell levels of combinations only including IFN-γ+ cells for four (IFN-γ, TNF-α, IL-2 and CD107a) and five (IFN-γ, TNF-α, IL-2, CD107a and PRF) functions. The medians with the interquartile ranges are shown. Each dot represents a patient. ROUT method was utilized to identify and discard outliers. *p < 0.05, **p < 0.01, ***p < 0.001. Mann-Whitney U test was used for groups’ comparisons. (Mild, n= 11; Severe, n= 11)

**Supplementary Figure 7. Combined SARS-CoV-2 specific T-cell response to N and S proteins in previously hospitalized and non-hospitalized subjects seven months after SARS-CoV-2 infection. (A)** Bar graphs represent the percentage of S plus N-specific CD4+ T-cell response. **(B)** Bar graphs represent the percentage of S plus N-specific CD8+ T-cell response. Each dot represents an individual. ROUT method was utilized to identify and discard outliers. *p < 0.05, **p < 0.01, ***p < 0.001,
****p < 0.0001. Mann-Whitney U test was used for groups’ comparisons. (H, n= 19; NH, n= 14)

**Supplementary Figure 8. Additional features of T-cell exhaustion and
SARS-CoV-2-specific T-cell response in previously hospitalized and non-hospitalized subjects seven months after SARS-CoV-2 infection.** **(A)** TIGIT expression in each CD8+ T-cell subset, **(B)** PD-1 expression in each CD4+ T-cell subset, **(C)** PD-1 expression in each CD8+ T-cell subset, in previously hospitalized and non-hospitalized subjects seven months after SARS-CoV-2 infection. **(D)** S-specific CM CD4+ T-cell levels of combinations only including TNF-α+ cells for three (IFN-γ,
TNF-α, and IL-2) (left panel) and four (IFN-γ, TNF-α, IL-2 and CD107a) functions (right panel). **(E)** N-specific EM CD4+ T-cell levels of combinations only including INF-γ+ cells for three (IFN-γ, TNF-α, and IL-2) (left panel) and four (IFN-γ, TNF-α, IL-2 and CD107a) functions (right panel). **(F)** N-specific CM CD8+ T-cell levels of combinations only including TNF-α+ cells for three (IFN-γ, TNF-α, and IL-2) (left panel) and four (IFN-γ, TNF-α, IL-2 and CD107a) functions (right panel). The medians with the interquartile ranges are shown. Each dot represents an individual. ROUT method was utilized to identify and discard outliers. *p < 0.05, **p < 0.01, ***p < 0.001,
****p < 0.0001. Mann-Whitney U test was used for groups’ comparisons. (H, n= 19; NH, n= 14)

**Supplementary Figure 9. Anti-S IgG levels are directly correlated with S-specific T-cell response in acute infection but this correlation is inverse seven months after SARS-CoV-2 infection in previously hospitalized patients. (A)** Correlation graphs between anti-S IgG levels and the percentage of N-specific EM CD4+ T-cells (left panel) and N-specific CD4+ EM IL-2+ T-cells in acute SARS-CoV-2 infection. **(B)** Direct correlation between anti-S IgG levels and the percentage of S-specific EM CD4+ T-cells (left panel) and S-specific CD4+ EM IL-2+ T-cells in previously hospitalized patients seven months after SARS-CoV-2 infection (right panel). Each dot represents an individual. ROUT method was utilized to identify and discard outliers. *p < 0.05,
**p < 0.01, ***p < 0.001, ****p < 0.0001. Spearman test was used for non-parametric correlations. (Acute, n= 31; Hospitalized, n= 17)

**Supplementary Figure 10. Anti-S IgG levels against endemic coronaviruses are associated with anti-S IgG levels against SARS-CoV-2.** Correlation matrix between anti-S IgG levels against SARS-CoV-2 and endemic coronaviruses (NL63, OC43, 229E and HKU1). Plasma sample was used at 1: 50 dilution. Blue color represents positive correlations and red color shows negative correlations. The intensity of the color indicates the R coefficient. ROUT method was utilized to identify and discard outliers. *p < 0.05, **p < 0.01, ***p < 0.001. Spearman test was used for non-parametric correlations. (Mild, n=18; Severe, n=19; HD, n=28)

**Supplementary Figure 11. S-specific T-cell response in HD to endemic coronaviruses is mainly mediated by IL-2 production.** **(A)** Bar graph represent the SE-specific T-cell response in each T-cell subset for each cytokine. **(B)** Correlation between S-Specific and SE-specific CM CD4+ IL-2+ T cell levels and **(C)** Correlation between S-Specific and SE-specific EM CD4+ IL-2+ T cell levels. Each dot represents an individual. ROUT method was utilized to identify and discard outliers. *p < 0.05, **p < 0.01, ***p < 0.001, ****p < 0.0001. Mann-Whitney U test was used for groups’ comparisons and Spearman test for non-parametric correlations. (HD, n = 33)

**Supplementary Figure 12. Schematic diagram of the gating strategy. (A)** Phenotyping of CD4+ and CD8+ T-lymphocyte subsets, including naïve (NAIVE); Total Memory (MEM), Central Memory (CM), Effector Memory (EM) and terminally differentiated memory (TEMRA) T cells. **(B)** Degranulation factor, CD107a. where negative control staining is on the left and positive sample on the right. **(C)** Gating of intracellular cytokine production, including interferon gamma (IFN-γ), interleukin-2
(IL-2) and tumor necrosis factor alpha (TNF-α). **(D)** Cytolytic enzime, mature perforin (PRF), where negative control staining is on the left and positive sample on the right. **(E)** Senescence, CD57+CD28-. **(F)** Exhaustion markers, T cell immunoreceptor with Ig and ITIM domains (TIGIT), where negative control staining is on the left and positive sample on the right; and programmed death 1 molecules (PD-1) and **(G)** Activation,
HLA-DR+CD38+.

**Supplementary Table 1. Characteristics of the study subjects.**

|  | Acute Infection | | | Discharged  (6-8 months after diagnosis) | | | Healthy Donors | | |
| --- | --- | --- | --- | --- | --- | --- | --- | --- | --- |
|  | **All**  **(n=37)** | **Mild**  **(n=18)** | **Severe**  **(n=19)** | **All**  **(n=33)** | **Previously**  **Hospitalized**  **(n=19)** | **Previously**  **Non Hospitalized (n=14)** | **All**  **(n=33)** | **Old HD**  **(n=19)** | **Young HD**  **(n=14)** |
| Age (years) | 71 [61.5 – 90] | 70 [57.75 – 76.5] | 72 [63 – 77] | 66 [58 – 74] | 71 [59 – 77] | 65.5 [56.75 – 71.5] | 62 [38.5 – 87] | 84 [71 – 90] | 38 [33.75 – 40.75] |
| Sex (Female sex), n (%) | 13 (35.1) | 6 (33.3) | 7 (36.8) | 12 (36.4) | 5 (26.3) | 7 (50) | 14 (42.4) | 10 (52.6) | 4 (28.6) |
| Oxygen Saturation (SatO_2_), (%) | 93 [91 – 97] | 96 [93 – 98.25] | 91 [87 – 95] | N/A | N/A | N/A | N/A | N/A | N/A |
| Time since hospitalization, (days) | 3 [2 – 21.5] | 2.5 [1 – 3.25] | 18 [3 – 28] | 201 [180.5 – 221] | 187 [173 – 193] | 221 [218 – 230.75] | N/A | N/A | N/A |
| Time since symptoms onset, (days) | 17 [7 – 31.5] | 7.5 [4 – 19.5] | 31 [17 – 37] | 208 [190 – 232] | 186 [195 – 202] | 232 [224.5 – 239.25] | N/A | N/A | N/A |
| Time hospitalized, (days) | 16 [7.5 – 29] | 8 [6.5 – 12.25] | 26 [17 – 36] | N/A | 19 [9 – 37] | 0 | N/A | N/A | N/A |
| Comorbidities, n (%)  Diabetes mellitus  Hypertension  Cardiovascular disease  Obstructive pulmonary disease  Malignancy | 29 (78.4)  12 (32.4)  25 (67.6)  11 (29.7)  4 (10.8)  4 (10.8) | 15 (83.3)  7 (38.9)  12 (66.7)  7 (38.9)  2 (11.1)  2 (11.1) | 14 (73.7)  5 (26.3)  13 (68.4)  4 (21.1)  2 (10.5)  2 (10.5) | 19 (57.6)  4 (12.1)  12 (36.4)  6 (18.2)  0  0 | 15 (78.9)  3 (15.8)  10 (52.6)  4 (21.1)  0  0 | 3 (21.4)  1 (7.1)  2 (14.3)  2 (14.3)  0  0 | N/A  N/A  N/A  N/A  N/A  N/A | N/A  N/A  N/A  N/A  N/A  N/A | N/A  N/A  N/A  N/A  N/A  N/A |
| Symptoms at admission (%)  Cough  Fever  Dyspnea  Anosmia  Diarrhoea  Muscle pain | 24 (64.9)  27 (73)  19 (51.4)  5 (13.5)  6 (16.2)  3 (8.1) | 11 (61.1)  11 (61.1)  7 (38.9)  0  3 (16.7)  1 (5.6) | 13 (68.4)  16 (84.2)  12 (63.2)  5 (26.3)  3 (15.8)  2 (10.5) | 22 (66.7)  25 (75.8)  16 (48.5)  6 (18.2)  9 (27.3)  6 (18.2) | 12 (63.2)  14 (73.7)  10 (52.6)  6 (31.6)  7 (36.8)  5 (26.3) | 10 (71.4)  11 (78.6)  6 (42.9)  0  2 (14.3)  1 (7.1) | N/A  N/A  N/A  N/A  N/A  N/A | N/A  N/A  N/A  N/A  N/A  N/A | N/A  N/A  N/A  N/A  N/A  N/A |
| Treatment during hospitalization; n (%)  Hydroxychloroquine  Lopinavir/Ritonavir  Beta Interferon  Corticoids  Remdesivir  Tocilizumab | 33 (89.2)  24 (64.9)  12 (20)  18 (48.6)  3 (8.1)  10 (27) | 15 (83.3)  8 (44.4)  2 (11.1)  4 (22.2)  3 (16.7)  1 (5.6Y) | 18 (94.7)  16 (84.2)  10 (52.6)  14 (73.7)  0  9 (47.4) | N/A  N/A  N/A  N/A  N/A  N/A | 19 (100)  16 (84.2)  7 (36.8)  12 (63.2)  0  10 (52.6) | N/A  N/A  N/A  N/A  N/A  N/A | N/A  N/A  N/A  N/A  N/A  N/A | N/A  N/A  N/A  N/A  N/A  N/A | N/A  N/A  N/A  N/A  N/A  N/A |

Categorical variables are expressed as number and percentages (%), and continuous variables are expressed as median (interquartile ranges [IQR]). N/A, not applicable. The different groups (acute infection, discharged patients and healthy donors) were age and sex matched. Chi-square test and a Mann-Whitney U test were used to compare categorical and continuous variables, respectively. Analysis by age; acute infection vs HD (p=0.423); discharged patients vs HD (p=0.700); Previously Hospitalized patients vs HD (p=0.635); Previously Non Hospitalized patients vs HD (p=0.907). Analysis by sex; acute infection vs HD (p=0.532); discharged patients vs HD (p=0.614); Previously Hospitalized patients vs HD (p=0.245); Previously Non Hospitalized patients vs HD (p=0.633). Severe participants were those who required Intensive Care Unit admission, or having ≥6 points in the ordinal scale score based on Beigel et al. (1) or death.

**Supplementary Table 2. Statistical description of variables from matrix correlation of figure S4.**

|  | **Acute** | | | **Mild Acute** | | | **Severe Acute** | | |
| --- | --- | --- | --- | --- | --- | --- | --- | --- | --- |
|  | **n** | **Median** | **IQR** | **n** | **Median** | **IQR** | **n** | **Median** | **IQR** |
| **TNF-α** | 36 | 10.51 | 5.920-12.790 | 17 | 7.9 | 5.095-11.950 | 19 | 10.97 | 6.250-13.110 |
| **IL-6** | 36 | 5.935 | 2.908-12.950 | 17 | 6.38 | 3.365-12.680 | 19 | 5.31 | 2.490-26.930 |
| **IL-8** | 36 | 10.4 | 5.683-20.030 | 17 | 7.69 | 4.770-19.490 | 19 | 10.94 | 8.340-21.650 |
| **IL-1β** | 36 | 1.15 | 0.545-1.748 | 17 | 1.25 | 0.355-1.935 | 19 | 1.06 | 0.6-1.600 |
| **MIP-1α** | 36 | 8.535 | 0.0375-16.630 | 17 | 10.81 | 0.150-21.930 | 19 | 6.88 | 0-15.110 |
| **MIP-1β** | 36 | 14.8 | 8.293-20.090 | 17 | 9.47 | 6.825-17.640 | 19 | 16.67 | 11.140-30.820 |
| **IFN-γ** | 36 | 36.03 | 25.380-54.960 | 17 | 38.47 | 28.030-60.970 | 19 | 35.8 | 21.090-42.130 |
| **CD25** | 37 | 2140 | 1252-3628 | 18 | 1637 | 842.400-3156 | 19 | 2276 | 1649-3883 |
| **IP-10** | 36 | 157.7 | 90.230-557.900 | 17 | 158.2 | 78.060-553.300 | 19 | 157.2 | 101.700-688.900 |

Continuous variables are expressed as median (interquartile ranges [IQR]).

**Supplementary Table 3. Anti-hCoV IgG antibodies OD (nm) raw data.**

|  |  |  | **hCoV IgG levels** | | | |
| --- | --- | --- | --- | --- | --- | --- |
| **Groups** | | **Donor** | **anti-NL63** | **anti-OC43** | **anti-229E** | **anti-HKU1** |
| **ACUTE INFECTION** | **MILD** | **M01** | 2.06050 | 2.96355 | 1.66870 | 1.67365 |
|  |  | **M02** | 3.11130 | 3.31210 | 2.77500 | 3.10020 |
|  |  | **M03** | 2.55805 | 3.44265 | 2.00490 | 3.00435 |
|  |  | **M04** | 2.07290 | 3.44095 | 2.06930 | 3.13145 |
|  |  | **M05** | 0.00000 | 0.00000 | 2.15405 | 3.23870 |
|  |  | **M06** | 3.10805 | 3.68865 | 3.06160 | 3.42290 |
|  |  | **M07** | 2.83610 | 2.58745 | 2.12805 | 1.53900 |
|  |  | **M08** | 1.91075 | 3.32160 | 2.14930 | 2.88035 |
|  |  | **M09** | 2.94100 | 2.24360 | 1.80360 | 1.54500 |
|  |  | **M10** | 1.44245 | 2.07775 | 2.41785 | 0.62415 |
|  |  | **M11** | 1.39020 | 3.23805 | 1.15420 | 1.91420 |
|  |  | **M12** | 2.51375 | 1.51760 | 2.36260 | 1.48930 |
|  |  | **M13** | 2.94055 | 3.35270 | 2.19175 | 2.77405 |
|  |  | **M14** | 1.24945 | 3.08910 | 1.94425 | 2.16265 |
|  |  | **M15** | 3.11155 | 3.24540 | 2.44125 | 2.61405 |
|  |  | **M16** | 2.63170 | 3.48915 | 2.76435 | 3.21230 |
|  |  | **M17** | 1.13435 | 3.23520 | 1.92100 | 2.87755 |
|  |  | **M18** | 2.57915 | 3.24715 | 2.42505 | 2.73475 |
|  | **SEVERE** | **S01** | 2.00120 | 2.89995 | 1.50810 | 1.75290 |
|  |  | **S02** | 0.91690 | 2.94775 | 1.38830 | 1.80175 |
|  |  | **S03** | 2.81545 | 3.50290 | 1.92365 | 2.99790 |
|  |  | **S04** | 1.90535 | 3.17535 | 1.43945 | 1.92655 |
|  |  | **S05** | 2.80295 | 3.12565 | 2.83170 | 2.70280 |
|  |  | **S06** | 2.09870 | 3.26910 | 2.88970 | 2.55740 |
|  |  | **S07** | 2.54025 | 3.27885 | 2.37210 | 2.69690 |
|  |  | **S08** | 1.15630 | 3.20725 | 1.68495 | 2.21965 |
|  |  | **S09** | 3.21110 | 3.47330 | 2.58010 | 3.14900 |
|  |  | **S10** | 2.53980 | 2.71260 | 2.93990 | 2.35875 |
|  |  | **S11** | 3.01670 | 3.40830 | 1.48055 | 2.84185 |
|  |  | **S12** | 3.01035 | 3.07690 | 2.59025 | 2.72425 |
|  |  | **S13** | 3.19805 | 3.40095 | 2.31100 | 2.97760 |
|  |  | **S14** | 2.30980 | 3.01010 | 0.61455 | 2.12250 |
|  |  | **S15** | 0.82135 | 3.44165 | 0.12615 | 3.15965 |
|  |  | **S16** | 1.65550 | 2.96715 | 0.80200 | 3.01000 |
|  |  | **S17** | 0.54665 | 2.92455 | 1.37010 | 2.45750 |
|  |  | **S18** | 2.52410 | 2.96640 | 2.15910 | 2.80970 |
|  |  | **S19** | 2.27360 | 2.84940 | 1.86385 | 2.31930 |

| **Continuation of Supp Table 3** | | | | | |
| --- | --- | --- | --- | --- | --- |
|  | | **hCoV IgG levels** | | | |
| **Groups** | **Donor** | **anti-NL63** | **anti-OC43** | **anti-229E** | **anti-HKU1** |
| HEALTHY DONORS | **HD01** | 2.78740 | 2.76360 | 2.16610 | 2.18175 |
|  | **HD02** | 1.56065 | 1.08055 | 1.85665 | 1.10430 |
|  | **HD03** | 3.46150 | 3.34870 | 2.18810 | 2.31240 |
|  | **HD04** | 3.32320 | 3.34310 | 2.34810 | 2.24415 |
|  | **HD05** | 2.84550 | 3.12610 | 2.78980 | 1.08700 |
|  | **HD06** | 2.48170 | 1.89540 | 2.35595 | 0.77970 |
|  | **HD07** | 3.14250 | 3.10555 | 3.07435 | 1.58460 |
|  | **HD08** | 2.41505 | 1.79415 | 2.76840 | 1.10670 |
|  | **HD09** | 1.41850 | 1.61420 | 1.38180 | 1.94045 |
|  | **HD10** | 1.76910 | 0.92290 | 1.50620 | 0.28585 |
|  | **HD11** | 2.18840 | 1.68420 | 2.74380 | 1.10005 |
|  | **HD12** | 0.43990 | 0.97100 | 0.64410 | 0.34825 |
|  | **HD13** | 3.23670 | 2.91245 | 2.68235 | 2.20570 |
|  | **HD14** | 2.71950 | 3.06770 | 2.50610 | 2.08350 |
|  | **HD15** | 3.21100 | 2.91520 | 2.00115 | 1.84450 |
|  | **HD16** | 3.00565 | 3.44410 | 2.60100 | 2.94880 |
|  | **HD17** | 3.23340 | 3.46235 | 2.74580 | 3.01430 |
|  | **HD18** | 3.07415 | 2.46865 | 2.18675 | 2.59050 |
|  | **HD19** | 2.17750 | 2.12810 | 2.48465 | 2.04760 |
|  | **HD20** | 2.74580 | 3.44920 | 2.43030 | 1.96845 |
|  | **HD21** | 3.45685 | 3.40485 | 1.90945 | 2.42975 |
|  | **HD22** | 2.90100 | 3.04070 | 3.00650 | 2.35845 |
|  | **HD23** | 2.71295 | 2.90215 | 3.09800 | 1.81250 |
|  | **HD24** | 3.29385 | 3.35510 | 3.07445 | 2.28220 |
|  | **HD25** | 2.32495 | 1.62785 | 1.64240 | 0.97640 |
|  | **HD26** | 3.10550 | 3.10445 | 1.88390 | 1.73530 |
|  | **HD27** | 3.30835 | 2.62560 | 2.83390 | 1.35510 |
|  | **HD28** | 1.92085 | 3.46900 | 2.09340 | 1.36995 |

**REFERENCES**

1. Beigel JH, Tomashek KM, Dodd LE, Mehta AK, Zingman BS, Kalil AC, et al. Remdesivir for the Treatment of Covid-19 — Final Report. N Engl J Med. 2020;383(19):1813–26.
